# Microbial-inoculated biochar combined with nitrogen mitigates salinity stress in rice by reducing salt accumulation and enhancing soil–plant interactions

**DOI:** 10.3389/fpls.2026.1751156

**Published:** 2026-03-24

**Authors:** Hafiz Muhammad Mazhar Abbas, Zongli Liu, Mohammad Nauman Khan, Haider Sultan, Asad Shah, Fahd Rasul, Ashar Tahir, Muhammad Nafees-Ur-Rehman, Lixiao Nie

**Affiliations:** 1 Sanya National Center of Technology Innovation for Saline-Alkali Tolerant Rice, School of Breeding and Multiplication (Sanya Institute of Breeding and Multiplication), Hainan University, Sanya, China; 2 Department of Agronomy, University of Agriculture Faisalabad, Faisalabad, Punjab, Pakistan; 3 School of Ecology and Environment, Hainan University, Haikou, China; 4 School of Life Sciences, Hainan University, Haikou, China

**Keywords:** bacterial biochar, fungal biochar, inoculation, microbial abundance, saline water

## Abstract

Microbial-inoculated biochar and N can enhance plant productivity under saline conditions through improving the chemical and biological properties of soil and the plants’ self-defense system. A pot experiment with 11 treatments was conducted to evaluate the effectiveness of rice husk biochar, with and without microbial (bacterial and fungal) inoculation, combined with nitrogen (N) in mitigating salinity stress (6.8 ds/m EC) in rice. Treatments included sole nitrogen (60 and 120 kg ha^-^¹), sole biochar (1%), microbial-inoculated biochar (fungal or bacterial), and their combinations with nitrogen at 60 and 120 kg ha^-^¹, applied under both saline and non-saline conditions. Two rice varieties were used: the salt-tolerant Shuang Liang You 138 (SLY138) and the salt-sensitive Jing Liang You 534 (JLY534). The experiment was conducted for 80 days. The combined application of bacterial-inoculated biochar (BB) and nitrogen fertilizer was more effective than rice husk biochar alone in improving soil microbial and chemical properties. Notably, bacterial-inoculated biochar combined with nitrogen 120 kg ha^─1^ (BB + N) treatments increased the abundance of pollution-degrading *Desulfobacterota* and salt-tolerant Actinobacterota. The enrichment of these microbial groups was associated with prolonged incubation time and reduced soil Na^+^ concentrations, which collectively contributed to improved rice growth. Compared to BC treatment, BB + N120 treatment produced higher proline, total soluble sugar (TSS), leaf water potential (Ψw), superoxide dismutase (SOD), peroxidase (POD), and catalase (CAT) in SLY138 by 120%, 167%, 30.23%, 50%, 68%, and 57%, respectively, under saline conditions and NH_4_^+^–N, OM, and bacterial and fungal community richness by 113.33%, 40%, 65.33%, and 186.25%, respectively. The salt tolerance of SLY138 may be correlated with the enhanced activities of SOD, POD, and CAT and more accumulation of proline and total soluble sugar. The findings of our study demonstrate the effectiveness of modified biochar applications in mitigating salinity stress and enhancing rice growth in saline environments.

## 1 Introduction

Rising global population necessitates an increase (87%) in staple crop (Nations, 2022). This population growth exacerbates environmental stresses (Karlen and Rice, 2015), potentially leading to decreased crop yields. Salinity is a major threat to crop production, affecting vast areas of global agricultural land (Wang et al., 2024). Global estimates indicate that hundreds of millions of hectares of land are affected by soil salinization worldwide, with more than 20% of irrigated agricultural land experiencing salinity constraints (Qadir et al., 2014; Huang et al., 2024). Saltwater intrusion and increased human activity worsen soil degradation, leading to saline soil with poor drainage and nutrient deficiencies, low vegetation cover, and ultimately, less productive land with reduced microbial activity (Amundson et al., 2015; Zhao et al., 2020).

Soil salinization and the use of saline irrigation water are among the most critical abiotic stresses threatening global food security by reducing crop productivity and degrading soil health (Wang et al., 2022; Hussein et al., 2023; Abbas et al., 2024b). In a study, it was also reported that saline water significantly deteriorates both the physicochemical and biological properties of soils due to excessive salt accumulation, particularly sodium (Na^+^), which leads to soil toxicity, reduced fertility, and impaired microbial activity (Abbas et al., 2024b). Irrigating with saline water enhances soil salinization, leading to a significant decline in agricultural soil quality and causing physicochemical deterioration, due to salt accumulation in the rhizosphere (Wang et al., 2011; El-Sayed et al., 2021). The excessive amounts of salt, primarily in the form of sodium (Na^+^), adversely affect soil properties, compromising the sustainability of agricultural production. Salinity significantly threatens rice (*Oryza sativa* L.), a staple food for over half of the world, due to its negative impacts on growth and yield. According to Hafeez et al. (2024), salinity negatively affects the wheat plant growth by disturbing the physiological attributes, biochemical processes of plants, and the availability of soil nutrients to plants. Moreover, it was also reported that salinity has adverse effects on the shoot and root morphology of brinjal plants (Jameel et al., 2024). Remediation of salt-affected soils could substantially increase the production of crops, like rice, that tolerate these conditions. Thus, the need to restore saline soils and effectively utilize these lands has become a pressing issue. Therefore, an effective measure that should be low-cost and eco-friendly is urgently needed to ameliorate soil salinity, promote plant productivity, and restore vegetation cover in coastal areas.

Biochar (BC), a carbon-rich product obtained through the pyrolysis of raw materials under high temperatures and limited oxygen, is being explored for its potential as a soil conditioner (El-Naggar et al., 2019; Wen et al., 2024) and for water pollution treatment (Qin et al., 2020). A study suggests that it may also be useful in mitigating salinity issues in soil by altering soil properties, with studies investigating various modifications to biochar to enhance this effect (Dahlawi et al., 2018). However, due to its high application rates, slow decomposition and slow release of nutrients in saline soil remain significant challenges (Wang et al., 2016; Mehdizadeh et al., 2020). On the other hand, Reddy et al. (2017) reported that contaminated soil often struggles to support the rapid growth of inoculated bacteria due to nutrient deficiency and competition from other microbes. Therefore, biochar may prove to be an ideal shelter for effective microorganisms because of its porous structure and affinity for microorganisms. BC possesses properties favorable for use as a bacterial carrier material, including high specific surface area, substantial internal porosity, and affinity for bacteria (Abbas et al., 2024a). Moreover, according to Fedeli et al. (2024); Cui et al. (2021), the synergistic effect of biochar and effective microorganisms offers an environmentally sustainable approach to promote plant growth and improve the remediation of saline and saline alkaline environment. Combined application of biochar and microbes effectively mitigated the salinity stress and improved the physicochemical properties and nutrient profile of soil, ultimately enhancing plant growth and yield (Zhang et al., 2019a).

The beneficial impact of biochar, especially in conjunction with N fertilizer, on agricultural productivity has been widely reported (Guo et al., 2004; Khan et al., 2019). Nitrogen (N) fertilizer combined with biochar can enhance biochar efficacy by promoting more complex microbial networks and potentially altering functions related to microbial symbiosis (Li et al., 2020). Moreover, Lu et al. (2015) reported that N fertilizer alters the soil microbial community, leading to a shift in biogeochemical cycles like SOC cycling and potentially enhancing biochar decomposition. To the best of our knowledge, the interactive use of microbial-inoculated biochar (BB and BF) and N to mitigate salinity effects on rice crop production has limited research during the past few decades.

Based on the synergistic interaction among biochar, microorganisms, and nitrogen (N), we hypothesize that:

1. Inoculated microbes within the biochar may survive more readily in saline conditions compared to free microbes in the soil.
2. The synergistic effect of microbial-inoculated biochar and N fertilizer may be higher than that of biochar and N application alone to accelerate the negative effects of saline water on plants and enhance their growth under saline stress by improving soil properties.
3. During synergistic effect of microbial-inoculated biochar and N fertilizer, N may assist microbial-inoculated biochar in alleviating salinity stress in paddy fields, promoting rice crop growth.

In this study, bacteria and fungi with higher sodium removal efficiency and potential for biochar decomposition were inoculated on biochar to prepare bacterial biochar (BB) and fungal biochar (BF). This study investigated (1) the combined effect of BB and BF with N fertilizer on remediating salinity in salt-affected sites, (2) the synergistic effect of microbial-inoculated biochar and N fertilizer on rice crop growth under saline conditions, and (3) the biological response of soil, focusing on microbial richness and microbial community structure. BB and BF were synthesized by physical adsorption method. Through this study, we will be able to provide an eco-friendly and sustainable way for the remediation of the salt effect from the soil and to improve crop production.

## 2 Materials and methods

### 2.1 Soil, biochar, and microbial strains

The soil used in this study was collected from a typical forest (*C. nucifera*) in Lingao County located in the northwestern part of Hainan Island, China. The physicochemical properties of pre-experimental soil were tested according to Xue et al. (2017). Coastal sandy soil was air-dried, sieved to 2 mm, and used for this study. Details on the physicochemical properties are presented in **Supplementary Table 1**. Rice-straw-derived biochar was commercially obtained from Henan Lize Environmental Technology Co., Ltd. The pH and nutrient status of biochar are presented in **Supplementary Table 2**. The biochar was passed through a 1.5-mm mesh before use. The bacterial strain *Mycobacterium* sp. (191574) and fungal strain *Penicillium* sp. (353380) was purchased from “BeNa Culture Collection” China (BNCC) and further multiplied in our lab. The bacteria and fungi were grown in “Lowenstein–Jensen medium” and “PDA media”, respectively, in a shaking incubator (120 rpm) at 30°C for 72 h.

### 2.2 Microbial biochar preparation and characterization

The *Mycobacterium* sp. and *Penicillium* sp. with higher salt removal efficiency were used to prepare bacterial- and fungal-loaded biochar (BB and BF, respectively). BB and BF were prepared by physical adsorption. Details about the preparation and characterization of BB and BF are presented in Supplementary Information Section 1.

### 2.3 Persistence of free and biochar-immobilized microorganisms under salt stress conditions

When comparing the survival rate of free-floating microbes to that of microbes immobilized in biochar in saline soil, it is crucial to ensure that both groups have the same initial concentration of microbes within the soil. The living cell numbers of *Mycobacterium* sp., *Penicillium* sp., BF, and BB were measured as described by Luo et al. (2021). Further details are presented in Supplementary Information Section 2.

### 2.4 Pot experiment and morphological traits of plants

A greenhouse experiment was carried out at Hainan University in Haikou, China (19.5664° N, 109.9497° E). Hainan is the second largest island in China, with an offshore area of approximately 2 million km², representing 42.3% of the nation’s total maritime area. The province’s annual precipitation is 2,176.7 mm, and the mean annual temperature is 25.1 °C. Plastic pots filled with 5 kg pot^─1^ soil were used, and these were laid out in a completely randomized block design with three replications. Following this, different combinations of biochemical materials were introduced to the soil alone or together; detailed information about the applied treatments is presented in the supplementary information (**Table 1**), and on the basis of morphological attributes, the best-performing treatments were selected for further analysis and presented in **Table 2**. Two rice cultivars were used in this study: the salt-tolerant Shuang Liang You 138 (SLY138) collected from Xike Agricultural Group Co., Ltd., and the salt-sensitive Jing Liang You 534 (JLY534) collected from Hunan Longping Hi-tech Seed Science and Technology Co., Ltd. These were subjected to two NaCl salinity levels: 0% (control) and 6.84 ds/m EC (salt stress) (Li and Stanghellini, 2001). Biochar (1%) was incorporated into the soil prior to pot filling, and nitrogen fertilizer (**Table 1**) was applied in three stages: at sowing, during the seedling stage, and 40 days after sowing (Sun et al., 2017). To promote early germination, seeds were soaked overnight and subsequently incubated for 36 h in a growth chamber. Six germinated seeds were sown in each pot, and after establishing a nursery, three plants per pot were sustained by thinning. Nitrogen was applied at rates equivalent to 60 and 120 kg N ha^-^¹, corresponding to 0.33 and 0.65 g urea per pot (5 kg soil), respectively. Nitrogen was supplied in the form of urea (46% N) and applied in three equal splits: at sowing, tillering, and panicle initiation stages. The nitrogen application rate was kept constant across treatments, irrespective of biochar or microbial (bacterial and fungal) inoculation, to allow an assessment of biochar-mediated nitrogen use efficiency rather than nitrogen dose effects. Phosphorus was applied uniformly to all treatments at a rate equivalent to 120 kg ha^-^¹ as P_2_O_5_ and potassium at 130 kg ha^-^¹ as K_2_O. Both phosphorus and potassium were applied as basal doses prior to sowing. Biochar application did not contribute additional mineral nitrogen; its role was limited to improving nutrient retention, microbial activity, and soil physicochemical properties. Salt was applied in irrigated water after 20 days of sowing by making 0% and 6.84 ds/m EC saline solution until 6.84 ds/m EC salt level was maintained in pots irrigated with NaCl salt solution. The pots with 0% salt (control) were irrigated with tap water. The salt levels of pots were determined with a salinometer (WS-200 PLUS) with a time domain. The plants were harvested before the reproductive stage (80 days after sowing).

**Table 1:** Treatment plan for the experiment.

Treatments	Salt levels	Treatment name	Application rates
T1	0%	N60	Nitrogen (60 kg ha^-1^)
	0.40%	N60	Nitrogen (60 kg ha^-1^)
T2	0%	N120	Nitrogen (120 kg ha^-1^)
	0.40%	N120	Nitrogen (120 kg ha^-1^)
T3	0%	BC	Simple rice straw biochar
	0.40%	BC	Simple rice straw biochar
T4	0%	BF	Fungal-inoculated biochar
	0.40%	BF	Fungal-inoculated biochar
T5	0%	BB	Bacterial-inoculated biochar
	0.40%	BB	Bacterial-inoculated biochar
T6	0%	BC + N60	Simple rice straw biochar + nitrogen (60 kg ha^-1^)
	0.40%	BC + N60	Simple rice straw biochar + nitrogen (60 kg ha^-1^)
T7	0%	BC + N120	Simple rice straw biochar + nitrogen (120 kg ha^-1^)
	0.40%	BC + N120	Simple rice straw biochar + nitrogen (120 kg ha^-1^)
T8	0%	BF + N60	Fungal-inoculated biochar + nitrogen (60 kg ha^-1^)
	0.40%	BF + N60	Fungal-inoculated biochar + nitrogen (60 kg ha^-1^)
T9	0%	BF + N120	Fungal-inoculated biochar + nitrogen (120 kg ha^-1^)
	0.40%	BF + N120	Fungal-inoculated biochar + nitrogen (120 kg ha^-1^)
T10	0%	BB + N60	Bacterial-inoculated biochar + nitrogen (60 kg ha^-1^)
	0.40%	BB + N60	Bacterial-inoculated biochar + nitrogen (60 kg ha^-1^)
T11	0%	BB + N120	Bacterial-inoculated biochar + nitrogen (120 kg ha^-1^)
	0.40%	BB + N120	Bacterial-inoculated biochar + nitrogen (120 kg ha^-1^)

**Table 2:** Selected treatments on the basis of best performance in morphology of rice plants.

Treatments	Salt levels	Treatment name	Application rates
T3	0%	BC	Simple rice straw biochar
	0.40%	BC	Simple rice straw biochar
T4	0%	BF	Fungal-inoculated biochar
	0.40%	BF	Fungal-inoculated biochar
T5	0%	BB	Bacterial-inoculated biochar
	0.40%	BB	Bacterial-inoculated biochar
T9	0%	BF + N120	Fungal-inoculated biochar + nitrogen (120 kg ha^-1^)
	0.40%	BF + N120	Fungal-inoculated biochar + nitrogen (120 kg ha^-1^)
T11	0%	BB + N120	Bacterial-inoculated biochar + nitrogen (120 kg ha^-1^)
	0.40%	BB + N120	Bacterial-inoculated biochar + nitrogen (120 kg ha^-1^)

### 2.5 Morphological indicators of plants

Before harvesting the rice plants of both varieties, we measured their plant height using a ruler and determined the number of tillers manually. Plant fresh weight (g/pot) was measured after harvesting using a weighing balance. Then, fresh biomass was transferred into the oven for 48 h at 105°C to measure its dry weight using a weighing balance.

### 2.6 Microbial and chemical properties of post-harvest soil

Post-harvest soil was analyzed for changes in chemical properties and microbial populations. Rhizosphere soil samples were subjected to total genomic DNA extraction using a TGuide S96 Magnetic Sol/Stool DNA Kit. DNA quality and quantity were determined by gel electrophoresis and NanoDrop spectrophotometry. The V3–V4 hypervariable region of the bacterial 16S rRNA gene was amplified using the primer pair 338F (5′ACTCCTACGGGAGGCAGCA3′) and 806R (5′GGACTACHVGGGTWTCTAAT-3′), which were modified with Illumina index sequences. Both 16S primers were tagged with unique Illumina barcodes for deep sequencing. The PCR reactions were conducted under specific conditions, and the amplified DNA fragments (amplicons) were purified and quantified before being sequenced using paired-end sequencing technology on an Illumina NovaSeq6000 platform. The details about the analysis for the chemical properties of post-harvest soil are presented in Supplementary Information Section 3.

### 2.7 Changes in relative water content, leaf water potential, and membrane stability index

The relative water content (RWC) of the leaves was measured using the gravimetric method as described by Barrs and Weatherley (1962). Leaf water potential (Ψw) was determined using Psypro Water Potential Measurement System and was reported (─MPa), while the membrane stability index (MSI) was estimated following the protocol described by Sairam et al. (1997). Additional information is provided in Supplementary Information Section 4.

### 2.8 Photosynthetic performance

During full shine from 10:00 a.m. to 2:00 p.m., we measured the amount of chlorophyll by using SPAD meter (SPAD-502 from Minolta Co., Ltd., Japan). One plant per replication was randomly selected, and the SPAD values of three fully mature leaves were measured from top to bottom. To assess photosynthetic performance, including chlorophyll fluorescence (Fv/Fm), non-photochemical quenching (NPQ), photochemical quenching coefficient (qP), and photosystem II excitation pressure (1-qP), a portable DUAL-PAM-100: P700 & Chlorophyll Fluorescence instrument was used. The photosynthetic parameters were measured after dark adaptation of the leaves for 30 min, with measurements conducted under controlled light conditions (7,500 µmol (photon) m^–2^ s^–1^ for a duration of 0.7 s).

### 2.9 External morphology of the leaves by scanning electron microscopy

Based on the morphological findings, we selected rice leaves treated with biochar BC, BF, BB, BF + N120, and BB + N120 for further analysis using scanning electron microscopy (SEM). For each chosen treatment, we took three small, equal-sized samples (1 mm^2^) from the middle sections of the leaves. The samples were rinsed with distilled water before being examined under an electron microscope. To preserve the samples, they were immersed in 4% glutaraldehyde solution buffered with 0.2 M sodium phosphate (pH 6.8) for 6 h at 4 °C. After fixation, the samples were rinsed four times with a solution of 0.1 M sodium phosphate buffer, still maintaining a pH of 6.8. Following the rinses, the samples were washed with a series of diluted ethanol solutions. The samples were washed twice with isoamyl acetate before being freeze-dried. Small sections of the leaf samples were adhered to stubs using a double-sided tape. Then, the samples were coated with a thin layer of gold by using a sputtering machine, Ion Sputter Coater (J20) (Khan et al., 2021; Kong et al., 2013). Finally, we examined the external features (morphology) of leaves by using a scanning electron microscope (SEM) Verios G4 UC, Thermo Fisher Scientific, Inc. (USA), and the areas of stomatal pore width (st.p.W) were determined by using the software Image J.

### 2.10 Determination of malondialdehyde, antioxidant enzymes, and osmotic substances

The amount of malondialdehyde (MDA) in the samples was measured following a previously described procedure by Heath and Packer (1968). The activities of antioxidant enzymes SOD, POD, and CAT were measured by following the protocols reported by Chance and Maehly (1955); Kono (1978); Aebi (1984). The final results for all enzyme activities were expressed in units (U g^─1^ FW).

Proline content was determined using the method described by Bates et al. (1973). Approximately 300 mg of fully expanded functional leaf tissue was homogenized in 10 mL of 3% (w/v) aqueous sulfosalicylic acid and filtered. Then, 2 mL each of ninhydrin acid and glacial acetic acid were added to 2 mL of the filtrate, and the mixture was heated for 60 min. The absorbance of the toluene layer was measured at 520 nm using a spectrophotometer. Proline content was computed using spectromax and expressed as mg g^─1^ fresh weight.

Total soluble sugar (TSS) content was analyzed using glucose as a standard (Bates et al., 1973). Soluble sugar content was measured as mg g^─1^ FW, using Spectromax.

### 2.11 Determination of Na^+^ and K^+^ in leaves

Before measuring the sodium (Na^+^) and potassium (K^+^) levels in the leaves, the leaves were washed with ddH_2_O to remove any salt particle adhering to the surface of the leaves. The washed leaves were gently dried with tissue paper and then placed in an oven at 80 °C until completely dehydrated. The dried leaves were ground using a mechanical grinder (Retsch MM 400). A 0.1-g sample of the ground material was transferred to a 50-mL glass tube, followed by the addition of 0.2 mL of ddH_2_O and 5 mL of concentrated H_2_SO_4_. For further analysis, the samples were placed in a digestion instrument (LWY84B, Siping Institute of Electronics Technique, Siping, China). After 1.5 h of digestion, the samples were supplemented with 0.2 mL of 30% H_2_O_2_ and mixed for 30 min. The digestion tubes were then returned to the digestion unit and heated until the appearance of white fumes, indicating completion of the digestion process. Once the white smoke indicating complete digestion appeared, the samples were carefully removed from the digestion instrument and allowed to cool. To make a 50-mL solution, ddH_2_O was added in glass tubes. To measure Na^+^ and K^+^, a flame photometer (INESA, FP 640) was set with the standard curve according to the manufacturer. Leaf Na^+^ and K^+^ concentrations were determined using a calculation method described by Khan et al. (2024).

### 2.12 Statistical analysis

Statistical analyses were conducted using SPSS software version 8.1. Data were presented as means ± standard deviation (*n* = 3). Two-way and three-way ANOVAs, followed by LSD tests (*p* < 0.05), were used to compare the treatment effects. Pearson correlation analysis was performed using R software version 4.4.1. The correlation plot was constructed using various R packages, including corrplot, extrafont, viridis, dplyr, stats, ggplot2, corrplot, metan, and RColorBrewer.

## 3 Results

### 3.1 Characterization of biochar and microbial-loaded biochar

**Figure 1:**
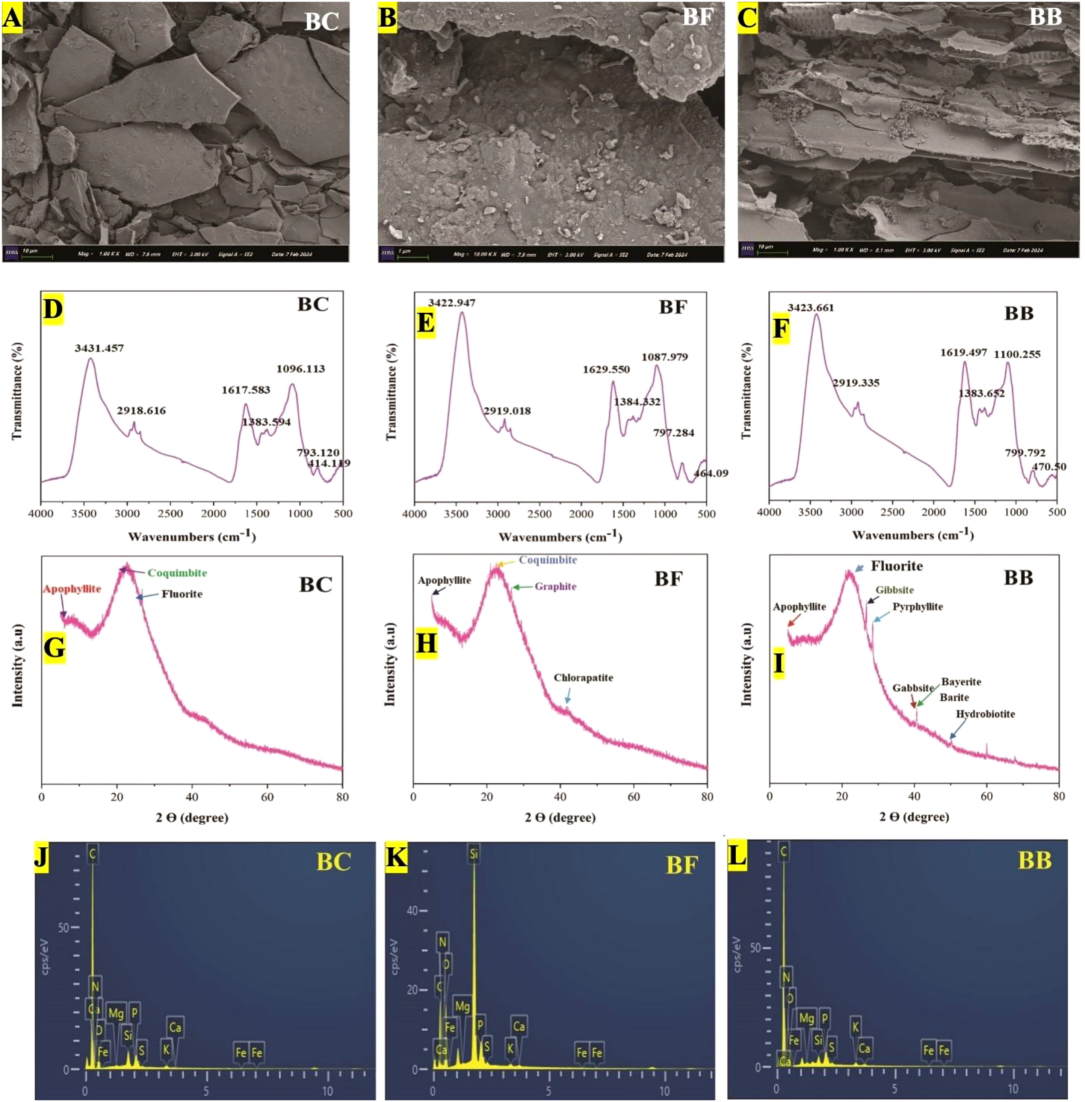
SEM, FTIR, XRD, and EDS analysis of biochar (BC), fungal biochar (BF), and bacterial biochar (BB), respectively. SEM image for BC **(A)**, SEM image for BF **(B)**, SEM image for BB **(C)**, FTIR for BC **(D)**, FTIR for BF **(E)**, FTIR for BB **(F)**, XRD for BC **(G)**, XRD for BF **(H)**, XRD for BB **(I)**, EDS for BC **(J)**, EDS for BF **(K)**, and EDS for BB **(L)**.

The SEM images showed that biochar has a rough and porous surface (**Figure 1A**), while microbial-loaded biochar was porous. This rough and porous surface might help the microbes stick and grow on the biochar. Many bacterial and fungal cells stick well to the biochar surface, either scattered out or clumped together (**Figures 1B, C**).

Moreover, the EDS analysis showed that C and O were the main elements found in all kinds of biochar. Na^+^ was not detected in these three kinds of biochar, whereas K^+^ was found in simple biochar (BC) and biochar modified by microbes, while biochar inoculated with *Mycobacterium* sp. has the most K^+^ (0.56 wt.%) than the others (**Figures 1J–L**). Furthermore, the EDS analysis also showed that other elements like N, O, S, and Mg were also found to be highest in bacterial-inoculated biochar (BB) than simple biochar BC. To better understand how the special molecules (functional groups) on biochar interact with *Mycobacterium* and *Penicillium* was analyzed by using FTIR spectroscopy (**Figures 1D–F**). Strong peaks were observed at 3,431.457, 3,422.947, and 3,423.661 in BC, BF, and BB, respectively. The second strongest and wide peaks were observed at 1,617.583, 1,629.550, and 1,619.497, respectively, in BC, BF, and BB. It needs to be emphasized that although all biochars (with and without immobilization) exhibited similar peaks, the structures were also not very different.

Different crystal structures can influence the properties of the biochar, which is important for its various applications. The X-ray diffraction patterns of rice straw biochar (BC), fungal biochar (BF), and bacterial biochar (BB) are presented in **Figures 1G–I**. The X-ray diffraction patterns of all three kinds of biochar (BC, BF, and BB) show a broad peak between 15° and 30° on the 2 theta (2θ) scale. This peak is assigned to the reflection of amorphous carbon, indicating that all kinds of biochar possess a disordered structure with randomly oriented aromatic carbon sheets. The XRD analysis of the bacterial biochar revealed the formation of a diverse range of mineral crystals and other inorganic materials, as evidenced by the appearance of new, narrower peaks within the 2θ range of 15°–40° and 40°–60° (**Figure 1I**). The X-ray diffraction pattern revealed some additional small peaks, and due to the overlapping nature of some minor peaks in the X-ray diffraction pattern, a qualitative approach was adopted for analysis. The XRD analysis of bacterial biochar revealed a prominent peak at 28°, indicating the presence of fluorite. Additionally, smaller peaks at 31° and 35° were attributed to gibbsite and pyrophylite. The XRD analysis of microbial biochars (BF and BB) demonstrated a diverse range of mineral components, including aluminum, carbon, silicon, calcium, magnesium, potassium, iron, and sulfur.

### 3.2 Aliveness of free microbes and the BF/BB immobilized microbes in saline soil

**Figure 2:**
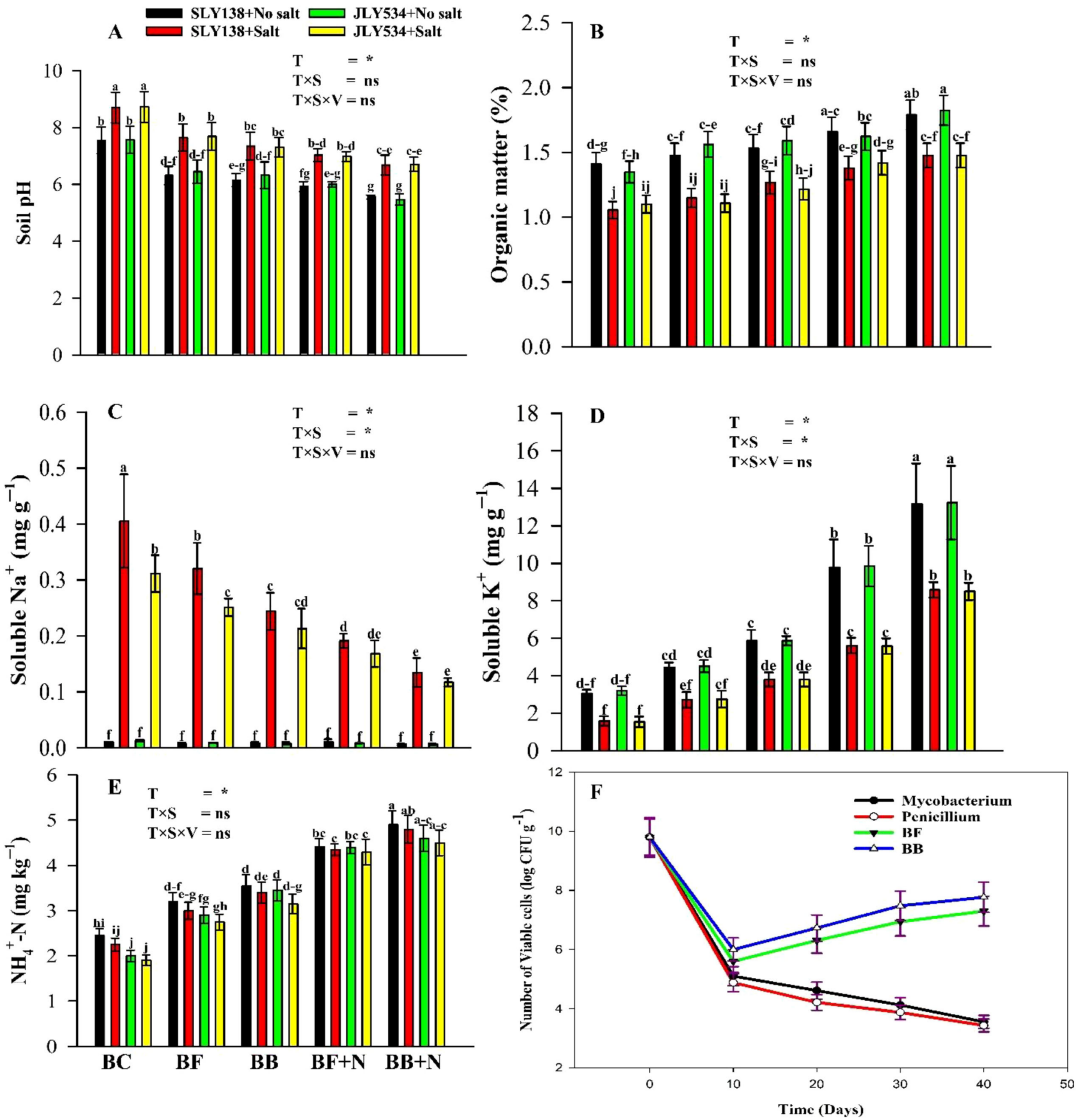
Synergistic effect of microbial-inoculated biochar and N fertilizer on the chemical properties of soil for both cultivars **(A)** pH of soil, **(B)** organic matter of soil, **(C)** soluble Na^+^ in soil, **(D)** soluble K^+^ in soil, **(E)** NH_4_^+^–N content in soil, **(F)** survival dynamics of free *Mycobacterium*, free *Penicillium*, mixed bacteria-loaded biochar (BB), and *Penicillium*-inoculated biochar (BF) in salt-contaminated soil. BC, simple biochar; BF, fungal biochar; BB, bacterial biochar; BF + N, fungal biochar and nitrogen; BB + N, bacterial biochar and nitrogen. The means that have the same letter do not differ substantially at *p* > 0.05 for a parameter. *, significant at *p ≤*0.05; ns, non-significant (*p* > 0.05); T, treatment; S, salt; V, variety.

The experimental results revealed a declining trend in free microbial populations, while immobilized microbial populations (BF/BB) exhibited a significant initial reduction in viable cell numbers within the first 10 days, thereafter transitioning to a phase of stable growth (**Figure 2F**). Overall, the microbes immobilized on biochar (BF and BB) exhibited greater survival rates in salt-contaminated soil compared to free-living cells. Notably, the density of viable cells followed the order: BB > BF > *Mycobacterium* > *Penicillium*. These results proved that our first hypothesis is correct.

### 3.3 Effect of microbial biochar on the chemical properties of post-harvest soil

The soil pH of saline soils is a direct indication of nutrient availability and crop growth. Overall, soil pH was increased by salinity and simple biochar (BC) compared to pre-experimental soil values. However, under saline conditions, a decrease in soil pH, by 23%, was reported in the BB + N120 treatment compared to the BC treatment. Additionally, a decrease in soil pH, by 20%, was also reported in the BF + N120 treatment compared to the BC treatment under saline conditions (**Figure 2A**). Salinity negatively affects soil OM, while all types of biochar (BC, BF, and BB) and N have positive effects on soil OM. However, BB + N120 and BF + N120 increased OM by 40% and 30%, respectively, compared to the BC treatment under saline conditions (**Figure 2B**).

As expected, application of saline water enhanced the soluble Na^+^ in soil as compared to non-saline conditions in the case of both varieties. However, BB + N120 decreased Na^+^ by 66%; similarly, BF + N120 decreased Na^+^ by 53% compared to the BC treatment under saline conditions (**Figure 2C**). In contrast to soluble Na^+^, salinity has negative effects on soluble K^+^ in soil. However, the soil soluble K^+^ content was increased by 440% and 252% in BB + N120 and BF + N120 than the lone application of BC under saline conditions (**Figure 2D**).

Compared with initial soil, the addition of biochar, microbial biochar (BF and BB), combined with N fertilizer resulted in elevated NH_4_^+^–N concentrations in both non-saline and saline conditions (**Figure 2E**). In contrast, salinity has negative effects on NH_4_^+^–N. However, BB + N120 and BF + N120 increased NH_4_^+^–N by 113% and 93%, respectively, compared to BC treatment under saline conditions.

### 3.4 Relative abundance of bacterial and fungal communities in saline conditions

**Figure 3:**
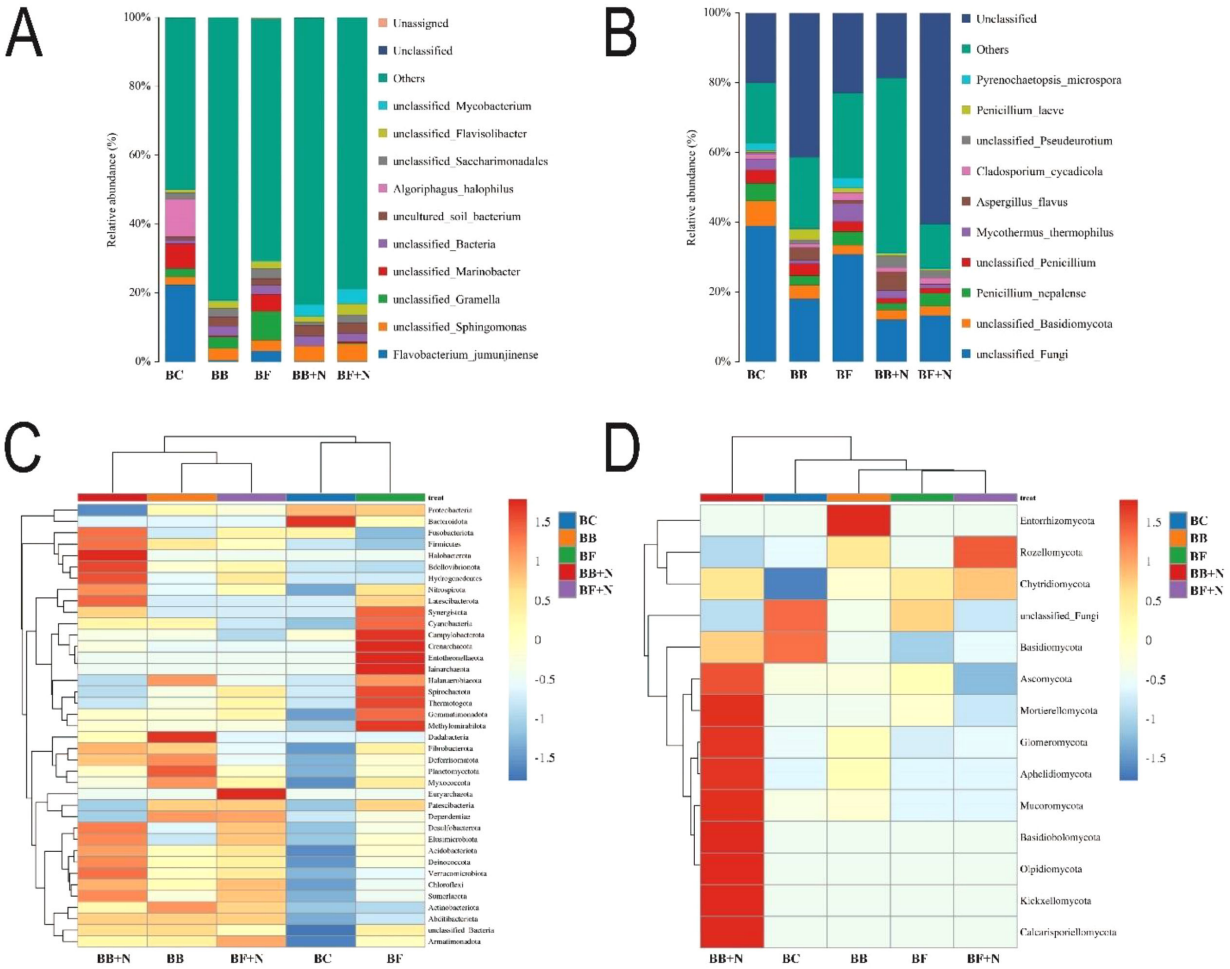
Synergistic effects of microbial-inoculated biochar and N fertilizer on the relative abundance of bacterial species **(A)** and fungal species **(B)**. Relative abundance of the top phylum of bacteria **(C)** and relative abundance of the top phylum of fungi **(D)** in saline soil.

Although salinity has negative effects on the biological properties of soil, compared to simple biochar (BC), microbial modified biochar (BF and BB) combined with N enhanced bacterial and fungal abundance in the rhizosphere saline soil of paddy fields (**Supplementary Figures 1A, B**). There were some large changes in the composition of bacterial and fungal communities (**Figures 3A–D**). A high relative abundance of three ecologically beneficial phyla of bacteria and one phylum of fungi *Nitrospirota*, *Actinobacteriota* and *Acidobacterota* and *Ascomycota* (fungi), respectively, was reported in the BB + N120 treatment. Both pollution-removal bacteria (*Desulfobacterota*) and salt-resistant bacteria (*Actinobacteriota*) were present in high concentrations in the microbial-biochar-treated soil (**Figure 3C**). Our analysis of the bacterial communities across all soil samples revealed *Proteobacteria*, *Actinobacteriota*, and *Chloroflexi* as the dominant phyla, and *Proteobacteria* emerged as the most abundant phylum in the ternary phase diagram (**Supplementary Figure 2**).

### 3.5 Effect of microbial biochar and N on MDA and antioxidant enzymes

**Figure 4:**
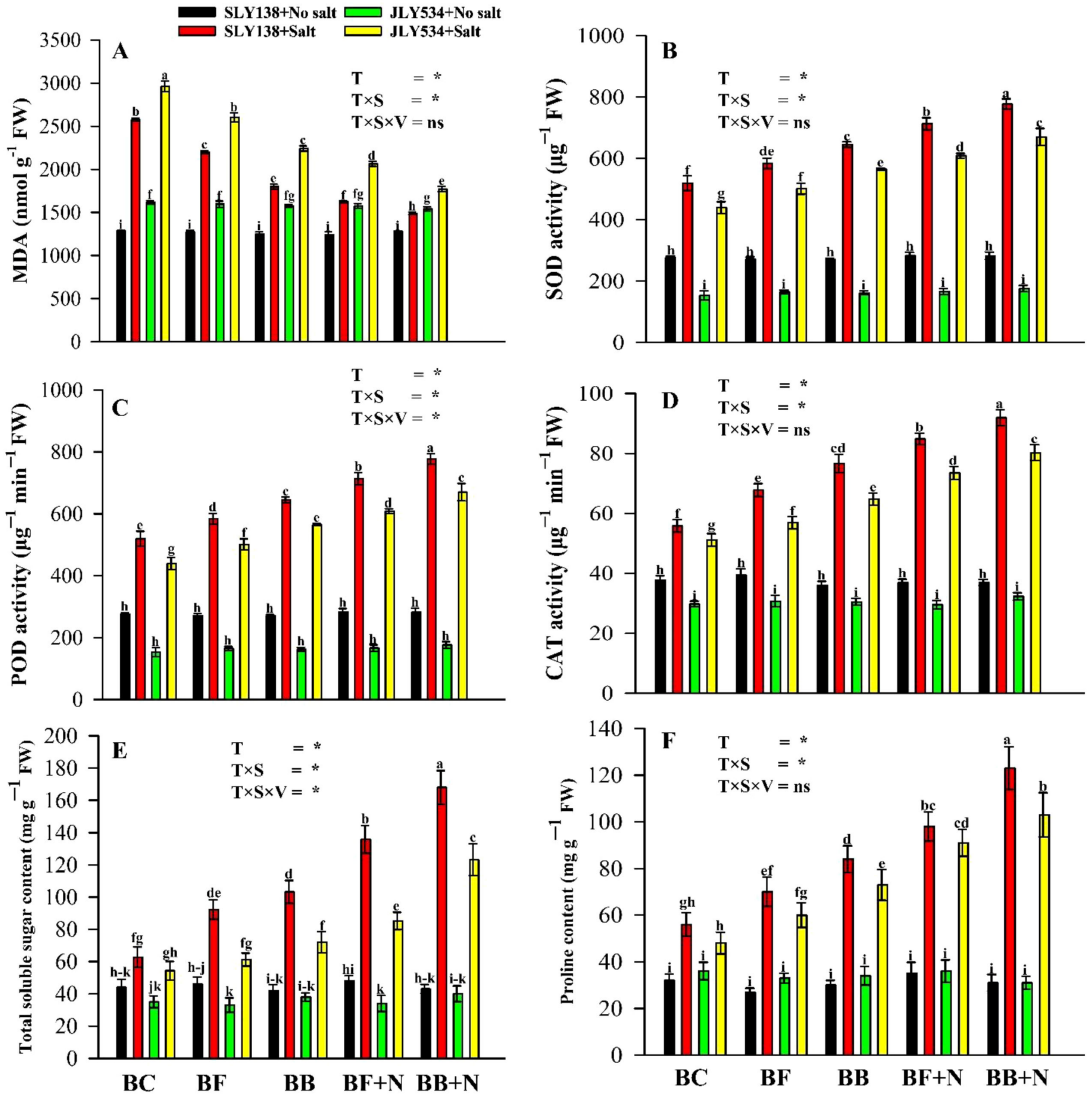
Synergistic effect of microbial-inoculated biochar and N fertilizer on the biochemical properties of rice leaves for both cultivars: **(A)** MDA activity in leaves, **(B)** SOD activity in leaves, **(C)** POD activity in leaves, **(D)** CAT activity in leaves, **(E)** total soluble sugar content in leaves, **(F)** proline content in leaves. BC, simple biochar; BF, fungal biochar; BB, bacterial biochar; BF + N, fungal biochar and nitrogen; BB + N, bacterial biochar and nitrogen. The means that have the same letter do not differ substantially at *p >*0.05 for a parameter. *, significant at *p ≤*0.05; ns, non-significant (*p* > 0.05); T, treatment; S, salt; V, variety.

Salt stress and the application of modified biochar significantly influenced malondialdehyde (MDA) concentrations within the leaves of the two investigated rice cultivars (**Figure 4A**). Notably, both rice varieties exhibited elevated MDA levels under saline conditions compared to the non-saline conditions. Under salt stress, SLY138 and JLY534 showed lesser MDA content in the BB + N120 treatment by 42% (1,490 nmol g^─1^ FW) and 40.18% (1,771.62 nmol g^─1^ FW), respectively, compared to BC treatment. Furthermore, JLY534 showed a higher MDA activity than SLY138 in the BC treatment under saline conditions. Notably, the synergistic effect of microbial biochars (BF and BB) and N fertilizer consistently demonstrated the strongest interactive effect, enhancing overall antioxidant activity in both rice varieties. There are significant variations between treatments in the activities of antioxidant enzymes, including superoxide dismutase (SOD), peroxidase (POD), and catalase (CAT) within the leaves of both rice cultivars under saline conditions (**Figures 4B–D**). SOD, POD, and CAT activities were increased in the BB + N120 treatment for SLY138 and JLY534 by 50%, 68%, and 64.5% and 52.4%, 68.33%, and 57%, respectively, compared to the BC treatment alone. Notably, SLY138 displayed a superior antioxidant response than JLY534 under saline conditions, as evidenced by elevated levels of key antioxidant enzymes catalase (CAT), peroxidase (POD), and superoxide dismutase (SOD).

### 3.6 Changes in osmotic substances

Proline and total soluble sugars (TSS) are key osmotic substances actively involved in rice osmoregulation under stressful conditions. However, proline content was enhanced in the BB + N120 treatment by 120% and 114% in SLY138 and JLY534, respectively, compared to the BC treatment (**Figure 4E**). Similarly, TSS was also higher by 168% in BB + N120 for SLY138 and 127% for JLY534 compared to the BC treatment (**Figure 4E**). Under the same growing conditions, the proline and TSS contents were higher by 17% and 16% in SLY138 compared with JLY534 (**Figure 4F**).

### 3.7 Membrane stability index, leaf water potential, and relative water content

**Figure 5:**
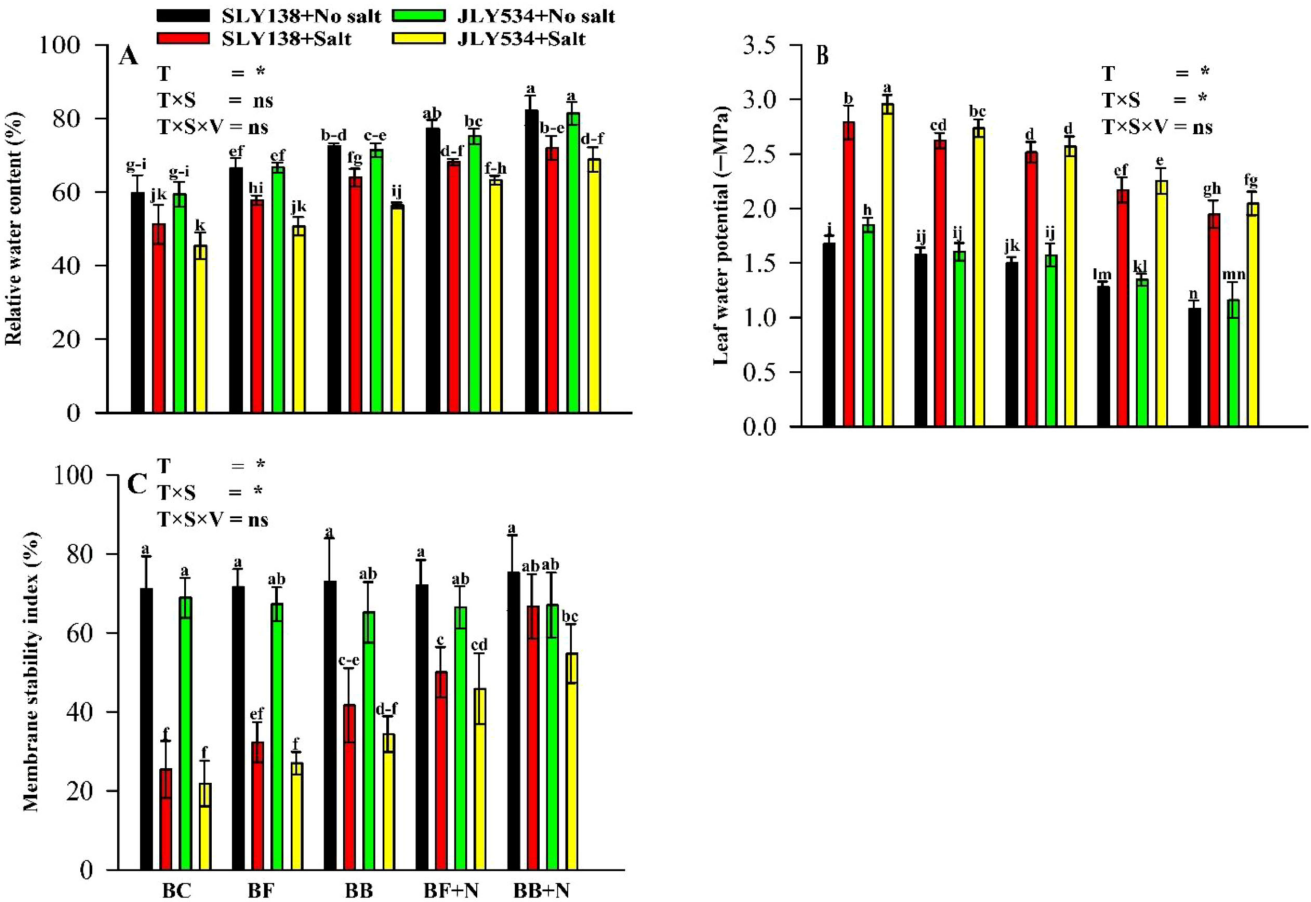
Synergistic effect of microbial-inoculated biochar and N fertilizer on RWC, Ψw, and MSI of rice plants. **(A)** Relative water content (RWC) in leaves, **(B)** water potential in leaves (Ψw), and **(C)** membrane stability index (MSI) in leaves. BC, simple biochar; BF, fungal biochar; BB, bacterial biochar; BF + N, fungal biochar and nitrogen; BB + N, bacterial biochar and nitrogen. The means that have the same letter do not differ substantially at *p >*0.05 for a parameter. *, significant at *p ≤*0.05; ns, non-significant (*p* > 0.05); T, treatment; S, salt; V, variety.

Salinity-induced osmotic stress, a primary cause of plant damage, compromises the integrity of cellular membranes as assessed by the membrane stability index (MSI). This damage leads to electrolyte leakage from cells. Salinity stress significantly reduced the membrane stability index (MSI), leaf water potential, and relative water content (RWC) in rice plants (**Figure 5**). However, BB + N120 increased the MSI, Ψw, and RWC values for SLY138 by 162.8%, 30.227%, and 150.6% compared to the BC treatment. Compared to the BC treatment, the MSI, Ψw, and RWC values for JLY534 in BB + N120 were higher by 40.5%, 30.78%, and 52.7%. Notably, under saline conditions, the MSI, Ψw% and RWC values were exhibited to be higher by 16.2%, 5.63%, and 13.5%, respectively, in SLY138 compared to JLY534 in the biochar (BC) treatment.

### 3.8 Effect of microbial biochar on the photosynthesis of rice plants under saline conditions

**Figure 6:**
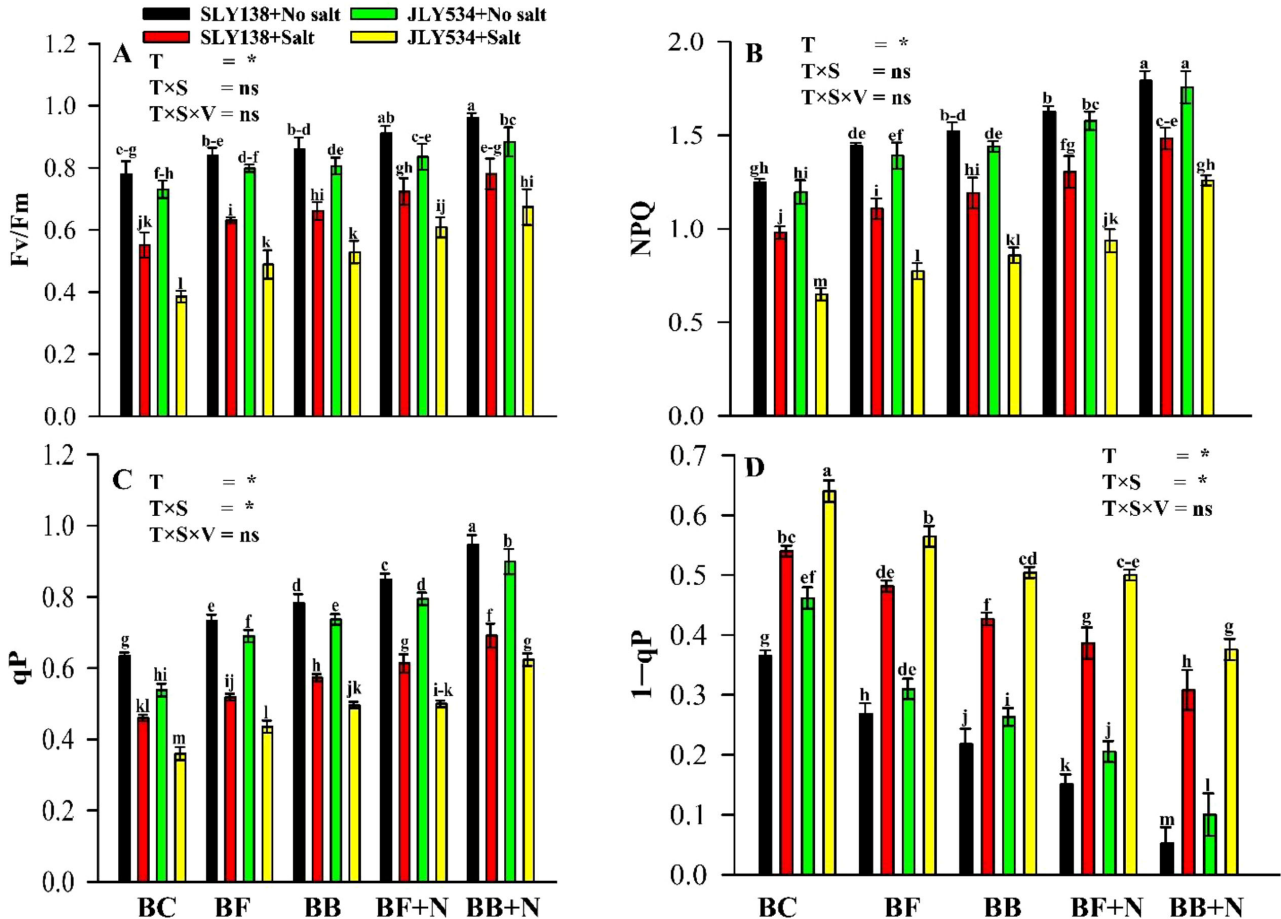
Synergistic effect of microbial-inoculated biochar and N fertilizer on the physiological properties of rice plants. **(A)** Maximum quantum efficiency (Fv/Fm), **(B)** non-photochemical quenching (NPQ), **(C)** photochemical quenching (qP), **(D)** photosystem II excitation pressure (1─qP). BC, simple biochar; BF, fungal biochar; BB, bacterial biochar; BF + N, fungal biochar and nitrogen; BB + N, bacterial biochar and nitrogen. The means that have the same letter do not differ substantially at *p >*0.05 for a parameter. *, significant at *p ≤*0.05; ns, non-significant (*p* > 0.05); T, treatment; S, salt; V, variety.

Our study found that under salt stress conditions, the BB + N120 treatment significantly improved the photosynthetic performance of rice plants compared to the biochar-only (BC) treatment (**Figure 6**). Furthermore, it was also reported that within the same rice variety and stress condition (non-saline or saline), all photosynthetic parameters—maximum quantum efficiency of PSII photochemistry (Fv/Fm), photochemical quenching (qP), non-photochemical quenching (NPQ), and photosystem II excitation pressure (1-qP)—exhibited significant differences between the various treatments. Under salt stress, the BB + N120 treatment significantly increased the Fv/Fm, NPQ, and qP values in SLY138 by 41.5%, 51.3%, and 50.4%, respectively, while corresponding increases of 75.1%, 94.0%, and 73.3% were observed in JLY534 (**Figure 6**). In contrast, under the BC treatment, the Fv/Fm, NPQ, and qP values in JLY534 were 30.2%, 33.7%, and 21.7% lower, respectively, than those observed in SLY138 under saline conditions.

### 3.9 Ultrastructure of stomata and stomatal pore width

**Figure 7:**
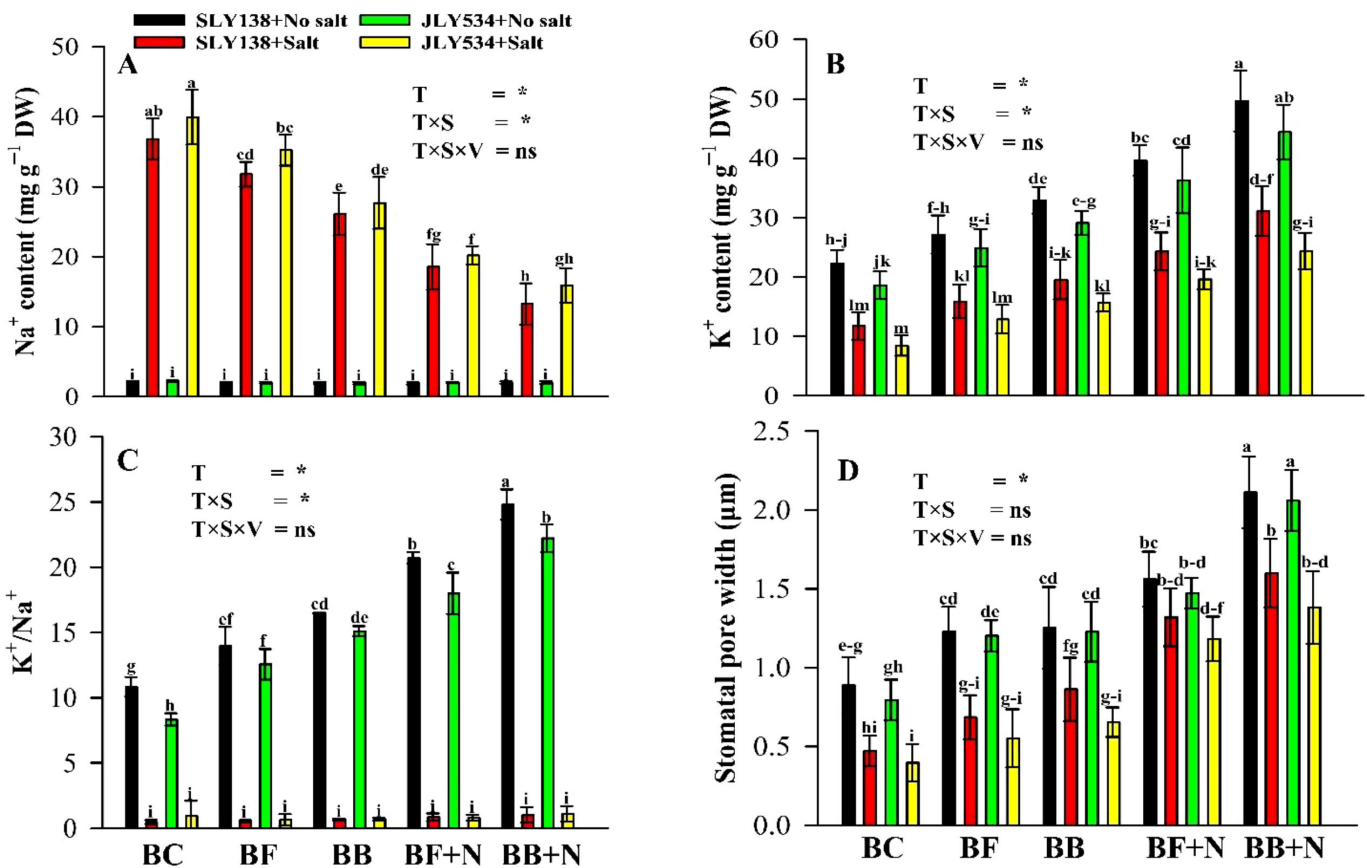
Synergistic effect of microbial-inoculated biochar and N fertilizer on Na^+^ and K^+^ content of rice plants. **(A)** Na^+^ content in leaves, **(B)** K^+^ content in leaves, **(C)** K^+^/Na^+^ in leaves, **(D)** stomatal pore width. BC, simple biochar; BF, fungal biochar; BB, bacterial biochar; BF + N, fungal biochar and nitrogen; BB + N, bacterial biochar and nitrogen. The means that have the same letter do not differ substantially at *p >*0.05 for a parameter. *, significant at *p ≤*0.05; ns, non-significant (*p* > 0.05); T, treatment; S, salt; V, variety.

Stomata are critical regulators of gas exchange in plants, directly influencing their growth and development. Given the crucial role of stomata in gas exchange and their observed influence on rice growth, we employed scanning electron microscopy (SEM) to analyze these structures in detail (**Supplementary Figure 3**). This analysis focused on stomatal pore width and potential ultrastructural changes in stomata across different treatments. Notably, salt stress exerted significant negative impacts on stomata, leading to a pronounced reduction in stomatal pore width (**Figure 7D**). Salinity stress decreased the pore width by 46% and 51% for SLY138 and JLY534, respectively, than in non-saline conditions. However, the BB + N120 treatment increased the stomatal pore width for SLY138 by 240.18% and for JLY534 by 249.9% compared to BC treatment alone under saline conditions (**Figure 7D**). Interestingly, SLY138 exhibited 19.7% higher stomatal pore width compared to JLY534 in the BC treatment under saline conditions. This observation suggests a potentially greater tolerance to salt stress in SLY138, possibly due to its ability to maintain wider stomatal apertures under salinity.

### 3.10 Influence of microbial biochar and nitrogen on K^+^/Na^+^ ratios in rice plants under salinity

Salinity increased the Na^+^ content in rice leaves (**Figure 7A**). All microbial biochar treatments and their combinations with N fertilizer performed significantly best compared to simple biochar in saline conditions. However, BB + N120 was the most effective treatment and decreased the Na^+^ content by 64% in SLY138 and 60% in JLY534 compared to the BC treatment. However, in SLY138, the Na^+^ content was 8% lesser than JLY534 in BC treatment under saline conditions. This difference between these two varieties is an evidence that SLY138 is more resistive to salt stress than JLY534. Not surprisingly, less K^+^ content was recorded in saline conditions compared to non-saline conditions (**Figure 7B**). However, K^+^ content was higher by 165% and 188%, respectively, for SLY138 and JLY534 in BB + N120 treatment compared to BC treatment. In varietal comparison, 39% more K^+^ content was recorded in SLY138 than JLY534 in the BC treatment under saline conditions. As there were big differences in the Na^+^ and K^+^ content for both varieties, BB + N120 performed better to maintain the K^+^/Na^+^ ratio under saline conditions (**Figure 7C**). Compared to the BC treatment, BB + N120 maintained a higher K^+^/Na^+^ ratio with 86.3% for SLY138 and 90.2% for JLY534, respectively, under saline conditions.

### 3.11 Morphological traits and SPAD values of rice plants

The agronomic characteristics of rice plants were significantly influenced by rice variety, salinity stress, biochar type, and nitrogen application (**Table 3**). Salt stress in the soil notably reduced the plant height (PH), number of tillers, plant fresh weight (PFW), and plant dry weight (PDW) of both rice varieties. However, fungal and bacterial biochar (BF and BB) application significantly enhanced these morphological parameters across both varieties, with a further increase observed with the application of higher nitrogen rates. Compared to the BC treatment, pH, number of tillers, PFW, and PDW in BB + N120 were higher by 172.95%, 273.684%, 452.11%, and 239.56%, respectively, for SLY138 under saline conditions (**Table 3**). These results proved that our second and third hypothesis are correct. Under saline conditions, pH, number of tillers, PFW, and PDW were higher for JLY534 in BB + N120 by 204.3%, 255.8%, 527.5%, and 510.13% compared to the BC treatment. Interestingly, JLY534 exhibited a greater reduction in these parameters compared to SLY138, suggesting a differential tolerance to salt stress between the two varieties. SLY138 grown under saline conditions has higher pH, number of tillers, PFW, and PDW by 28%, 48.3%, 28.65%, and 123.6%, respectively, in the BC treatment. Additionally, chlorophyll content (SPAD) was 8.4% higher in SLY138 under these conditions.

**Table 3:** Effect of microbial-inoculated biochar and N fertilizer on the morphology and SPAD of rice crop under saline conditions.

Treatments	Salt (%)	Plant height (cm)		Plant fresh weight (g pot^─1^)		Plant dry weight (g pot^─1^)		No. of tillers/pot		SPAD	
		SLY138	JLY534	SLY138	JLY534	SLY138	JLY534	SLY138	JLY534	SLY138	JLY534
N60	0	33.7 u	29.1 vw	20.9 v	36.3 w	18.6 q–s	17.6 st	12.3 uv	10 vw	20.9 tu	19.8 uv
	0.4	22.7 x	17.8 y	16.2 y	18.9 z	10.9 v	4.9 x	8.5 wx	5.8 x	16.2 wx	14.9 x
N120	0	49.5 no	46.6 o–q	24.4 t	55.2 t	24.8 n	21.8 op	19.8 o–q	17.3 q–s	24.4 o–q	23.8 p–r
	0.4	38 tu	33.5 uv	21.1 vw	30.6 x	15.9 tu	8.2 w	12 uv	11.8 uv	21.1 s–u	19.1 uv
BC	0	41.2 r–t	38.5 st	21.3 u	39.6 vw	20.3 p–r	18.1 p–t	16.5 r–t	13.5 tu	21.3 r–u	20.8 u
	0.4	33.8 u	27.4 w	17.5 xy	22.9 z	12.5 v	5.7 x	9.5 vw	8.5 wx	17.5 vw	15.6 wx
BF	0	61.1 j	58.9 j–l	29.4 kl	110.6 l	29.1 kl	28.2 lm	25.3 k–m	22.3 m–o	29.4 i–k	28.8 j–l
	0.4	49.6 no	47.2 op	26.2 p	87.9 q	21.1 p	12.5 v	17.8 q–s	15.8 st	26.2 m–p	23.6 p–s
BB	0	73.6 f	71.4 f–h	34.4 i	123.5 j	35.1 hi	33.5 ij	29 g–j	27.8 h–k	34.4 fg	33.5 g
	0.4	60.6 jk	56.1 k–m	29.7 m	101.1 no	25.2 n	16.1 tu	22.8 m–o	18.5 p–s	29.7 ij	27.6 j–m
BC + N60	0	55.8 lm	53.1 mn	26.8 r	72.3 s	28.6 lm	26.2 mn	23.8 l–n	21.3 n–p	26.8 l–o	26.9 k–o
	0.4	43.9 pr	42.7 qs	24.9 t	41.3 uv	19.7 ps	10.7 v	16.5 r–t	15.8 st	24.9 n–q	23.4 q–t
BC + N120	0	68.5 g–i	67 hi	33.2 mn	99.1 o	33.6 hj	31.4 jk	27.8 h–k	25.3 k–m	33.2 gh	32.9 gh
	0.4	55.2 lm	52.9 mn	27.5 q	75.9 rs	24.1 no	14.9 u	20 o–q	19 p–r	27.5 j–n	25.8 m–q
BF + N60	0	82.3 cd	80.5 cd	38.8 f	137.5 gh	40.9 f	37.6 g	33 ef	31.8 fg	38.8 de	38.5 de
	0.4	71.1 f–h	65.7 i	33.1 j	116.8 k	30.2 kl	20.9 pq	26.8 j–l	22.8 m–o	33.1 gh	31.4 hi
BF + N120	0	92.2 b	90.7 b	43.7 cd	151.8 e	45.6 cd	42.9 ef	37 cd	35 de	43.7 bc	44.2 bc
	0.4	78.7 de	72.1 fg	38.3 h	127 ij	34.7 hi	26.5 mn	30 f–i	26.5 j–l	38.3 de	36.6 ef
BB + N60	0	94.7 b	93.2 b	45.2 c	154 de	47.9 bc	44.4 de	38.3 bc	36.5 cd	45.2 b	45.2 b
	0.4	82.5 cd	74.8 ef	39.4 g	129.8 i	36 gh	28.4 lm	31.3 fg	27 i–k	39.4 d	37.1 de
BB + N120	0	107.5 a	103.1 a	51.7 a	169.4 b	52.8 a	49.2 b	42.5 a	40.8 ab	51.7 a	50.5 a
	0.4	91.9 b	83.2 c	45.6 e	143.9 f	42.3 ef	34.6 gh	35.5 c–e	30.3 f–h	45.6 b	42.2 c
T		*		*		*		*		*	
T × S		*		*		*		ns		*	
T × S × V		ns		ns		ns		ns		ns	

Means that have the same letter do not differ substantially at *p* > 0.05 for a parameter.

*, significant at *p ≤*0.05); ns, non-significant (*p* > 0.05); T, treatment; S, salt; V, variety.

### 3.12 Pearson correlation

**Figure 8:**
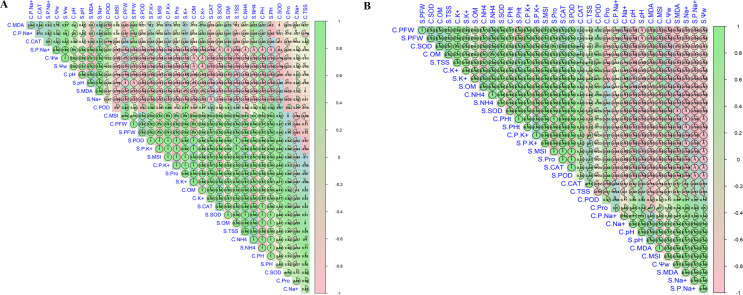
Pearson correlation test for both varieties: **(A)** SLY138. **(B)** JLY534.

The results showed that the morphological attributes (PFW, PH, PDW, tillers, and SPAD) of rice plants have a positive correlation with soil OM, available nitrogen NH_4_^+^–N, soluble K^+^ in soil, and also with antioxidant enzymes, including SOD, POD CAT, TSS and proline, for both cultivars under saline conditions (**Figures 8A, B**). However, the above-mentioned attributes have a negative correlation with soil pH, soluble Na^+^ in soil, total Na^+^ in plants as well as MDA activities in plants for both cultivars under saline conditions.

## 4 Discussion

### 4.1 Biochar as a host for inoculated microbes to survive in degraded soil

The decline in free microbial populations is due to the toxic effect of Na^+^ and Cl^─^ in soil and also due to the sudden change in pH after salt and biochar addition. In their study, Liu et al. (2020) also stated that a sudden change in soil pH (Wei et al., 2022) and other chemical properties can cause a decline in microbial population. The initial decrease in bacteria and fungi density (**Figure 2F**) was likely caused by the microbes’ inability to adapt to the harsh, salty environment. However, the latter increase in inoculated microbial population could be due to (1) the microorganisms inoculated within the biochar, which slowed their release into the surrounding stressful environment (Wei et al., 2022) and (2) the reproduction of bacteria and fungi that had adapted to the salty environment within the BF/BB carrier (Abbas et al., 2024a) (**Figure 2F**). These results suggest that biochar played a beneficial role in microbial proliferation. In many studies, it was reported that biochar provides a suitable habitat and essential nutrients for the microorganisms, ultimately stimulating their growth (El-Naggar et al., 2019; Qi et al., 2021). Additionally, the biochar may absorb and reduce the availability of salts (Na^+^ and Cl^─^) to microorganisms (He et al., 2019; Wang et al., 2019). The results also showed that bacteria and fungi could not maintain a similar survival rate on biochar. The relatively lower colonization of fungi on biochar compared to bacteria is due to the high levels of mineral elements and organic compounds in biochar (Lehmann et al., 2011) and also to the abrupt changes in soil pH (Chen et al., 2013). Interestingly, when microbes were applied in a certain range of saline environment, salt-resistant bacteria grow even better (Rath et al., 2019). Moreover, among inoculated microbes, bacteria are more resistant to salinity than other microbes (Xing et al., 2024). It is suggested that genetic differences in the physiology of the microbes may also play a role, directly or indirectly, to affect their survival and reproduction in the salty environment.

### 4.2 Characterization of biochar

The colonization of microbes like bacteria and fungi on biochar mainly depends upon their physiological properties and the physicochemical properties of their colonized material (Tu et al., 2020). The mechanical strength, high surface area, and porous structure of biochar make it an ideal carrier for microbes, providing a conducive environment for their growth (Wu et al., 2020). Additionally, biochar possesses functional groups on its surface (hydroxyl, carboxyl, phosphate, amine, and sulfhydryl) that can interact with similar groups on bacteria and fungi (Wu et al., 2021). These interactions, including ionic bonding, hydrogen bonding, and van der Waals forces, contribute to the adhesion of microbes to biochar. Elemental analysis showed that biochar inoculated with bacteria has more contents of O, Mg, and S (**Figure 1L**).

Peaks near 3,408.64 cm^─1^ indicate the presence of hydroxyl (-OH) groups in polysaccharides and amino (-NH) groups in proteins, which are common components of biochar (Dhal et al., 2010). The peak at 2,923.05 cm^─1^ corresponds to the stretching vibrations in the carbon–hydrogen (C–H) bonds of alkyl groups present in the biochar (Karthik et al., 2017). The wide range of peaks at approximately 1,651.74 cm^-1^ indicates the bending vibrations within amide I and amide II bonds. These bonds are commonly found in proteins and other molecules containing peptide groups (Wu et al., 2019). In all three samples, the strong peak observed at ≤1,100 cm^─1^ points to the presence of carbon–oxygen (C–O) bonds in polysaccharides within the biochar. In the curves, this peak might also be due to the presence of orthophosphate (1,100–1,080 cm^─1^), a type of phosphate molecule. The peak at 800 cm^-1^ corresponds to the stretching vibrations in the carbon–hydrogen (C–H) bonds of aromatic molecules present in the sample. Different types of microbes can have varying effects on the structural and chemical composition of biochar. In their study, Novak et al. (2010) reported that *Mycobacterium* sp. was capable of hydroxylating aromatic compounds present in biochar.

### 4.3 Impact of microbial-modified biochar and nitrogen on soil physicochemical and biological properties

Nutrient availability and plant growth directly depend upon the soil pH (Zhang et al., 2023). Saline water increased the soil pH as it is the cause of reducing reactions and consumes H^+^ (Van Tan and Thanh, 2021). In our study, both kinds of microbial-modified biochar (BF and BB) combined with nitrogen significantly reduced the soil pH by 23% and 20%, respectively, even under saline conditions compared to unmodified biochar (**Figure 2A**). In their study, Tu et al. (2020) reported three possible reasons of decrease in soil pH: (1) the production of acidic metabolites by native and inoculated microbes during the decomposition of soil organic matter or plant residues plays a significant role, (2) inoculated microbes (*Mycobacterium* sp. and *Penicillium* sp.) might release small organic acids as waste products that may cause a decrease in soil pH—many studies reported that inoculated microbes can decrease soil pH (Qi et al., 2022; Abbas et al., 2024a), and (3) the proliferation of acid-producing native microbes in the soil, potentially facilitated by inoculated microbes, which leads to a reduction in soil pH. Moreover, when treated with N-containing fertilizer, the slight decrease in soil pH could be caused by the release of protons (H^+^) during the nitrification process in soil (Sharma et al., 2022). In contrast to the BF and BB treatments, the BC treatment increased the soil pH (**Figure 2A**), which was likely due to the production of alkaline substances in the biochar during the pyrolysis process (Yuan et al., 2011).

Soil salinity has negative effects on C dynamics as it can decrease the decomposition rate of SOC by inhibiting microbial activity by reducing the osmotic potential in the soil solution and causing ionic toxicity (Abbas et al., 2024b). The addition of biochars (BC, BF, and BB) and N increased the soil organic matter (OM) compared to the BC treatment (**Figure 2B**). However, microbial mineralization was the dominant way for the decomposition of OM in soil. In many studies, it was reported in detail that the decomposition process of biochar could provide an energy source for inoculated and activated native microbes (Lian and Xing, 2017). According to Ameloot et al. (2013), microbial mineralization of biochar in soils typically consist of two stages, a fast-degrading stage and slow-degrading stage. Labile fractions are broken down quickly by microbes, and condensed aromatic ring structures degrade much more slowly. Nitrogen also enhanced SOM by fasting biochar’s decomposition and also native soil SOM by changing the soil microbial community. As a result, there was a shift in the functioning of the biogeochemical cycles (Lu et al., 2015). As nitrogen can change the soil microbial community, it could change the inoculated microbial community in biochar.

Soluble sodium (Na^+^) in the soil increased under saline conditions (**Figure 2C**). However, biochar might help reduce the amount of Na^+^ in saline soil due to a few reasons caused by the biochar itself. Biochar can accelerate the leaching of sodium (Na^+^) from the soil profile by improving the physical properties of the soil (Jin et al., 2024). Additionally, the release of cations such as calcium (Ca^2+^) and magnesium (Mg^2+^) can facilitate the displacement of sodium from cation exchange sites and the soil solution (Dahlawi et al., 2018), and characterization of our biochars show that these bivalent cations are high in bacterial biochar (BB) compared to BF and BC (**Figure 1L**). Inoculated and activated native microbes may assist biochar in reducing the sodium (Na^+^) levels in the soil through bioaccumulation. In response to the increased external salinity, some halophilic microbes can absorb salt ions to maintain cell osmotic pressure, potentially exporting sodium ions in the process (Zhang et al., 2023). As a result of these adjustments by the microbes and biochar, the levels of Na^+^ ions in the soil decreased more in BB + N120 treatment. Biochar increased the potassium (K^+^) levels in the soil due to its negative surface charge and porous structure, which retain nutrients under saline and water-stressed conditions (Liu et al., 2016).

Plants absorb nitrogen in two forms: ammonium (NH_4_^+^–N) and nitrate (NO_3_^─^-N) (Zhao et al., 2016). In our study, BB + N120 was the most effective approach to increase NH_4_^+^–N (**Figure 2E**). Previously, it was reported by Dendooven et al. (2010); Sun et al. (2017) that the alkaline nature of soils would favor the transformation of NH_4_^+^–N phase into solution phase (NH_3_ phase). As in our study, the pH of both conditions (non-saline and saline) was acidic to neutral (**Figure 2A**) and is favorable for the NH_4_^+^–N contents in soil. According to Chen et al. (2013), the biochar’s positive effects on NH_4_^+^–N are attributed to its adsorption capacity, which arises from electrostatic interactions and pore filling (Chen et al., 2013; Zheng et al., 2013). Additionally, microbes enhanced the concentration of NH_4_^+^–N through soil mineralization processes, suggesting their ability to fix nitrogen in the soil (Pan et al., 2022).

Similar to the impact on physicochemical soil properties, high salinity levels adversely affect the population size, activity, and diversity of soil microbes (Yao et al., 2023). However, microbial-inoculated biochar has been shown to increase the microbial abundance in degraded soils (Qi et al., 2021). According to Bello et al. (2021), an increase in soil bacterial abundance fostered the development of symbiotic relationships, leading to enhanced nutrient cycling. The observed increase in bacterial and fungal abundance might be due to the reduced competition with indigenous microbes resulting from the biochar-adapted consortium as biochar provides a favorable niche to inoculated microbes (Wei et al., 2022). Furthermore, microbial inoculation on biochar resulted in superior community richness and diversity compared to the other treatments (Qi et al., 2022) because inoculated microbes likely utilize the labile fractions of biochar as a readily available energy source, promoting short-term microbial activity within the community. Soil microbial communities are well recognized as complex networks, characterized by ecological interactions and niche partitioning among member organisms (Zhang et al., 2018). The adaptability of bacteria to changing environmental conditions can be assessed by examining their diversity and functional characteristics (Cheng et al., 2018). Notably, *Proteobacteria*, *Actinobacteriota*, and *Chloroflexi* emerged as the most abundant phyla (**Supplementary Figure 2**), consistent with their well-established tolerance to saline environments (Xu et al., 2020; Gu et al., 2022; Sun et al., 2023; Zhang et al., 2023). This prevalent group plays a vital role in nitrogen cycling within saline soils. Biochar’s abundant functional groups may indirectly alter the microbial community structure by immobilizing Na^+^ and Cl^─^ in saline soil. Among fungal phyla, *Ascomycota* is the most abundant phylum in saline conditions (**Figure 3D**). *Ascomycota* is a saprotrophic fungi and the most important organisms involved in the degradation and decomposition of OM (Shah et al., 2016). *Nitrospirota* and *Actinobacteriota* are the phyla of important microorganisms involved in nitrification process (Shi et al., 2018; Samaddar et al., 2019). Biochar facilitated the nitrification process by enhancing the microbial community and activity of nitrifying bacteria within the soil (Hou et al., 2021). Microbial biochar positively influenced both pollution-removing bacteria (*Desulfobacterota*) and salt-resistant bacteria (*Actinobacteriota*) (**Figure 3C**). Numerous studies have highlighted the role of these bacteria in improving purification performance through their impact on bacterial community composition (Ajibade et al., 2023; Huang et al., 2023). However, applying biochar, particularly microbial-inoculated biochar with nitrogen, improved the physicochemical and biological properties of soil. This suggests that microbial (bacterial and fungal) inoculation enhances biochar’s impact on soil microorganisms compared to simple biochar, which primarily influences the chemical properties of soil and ultimately enhances the available nutrient levels.

### 4.4 Microbial-modified biochar with N modulated MDA, antioxidant enzymes, and osmolytes in rice plants under saline conditions

Salinity affected the biochemistry of rice plants (**Figure 4**). When plants experience salt stress, their essential biochemical processes are inhibited. Salinity causes oxidative stress in plants by generating ROS (You and Chan, 2015). In the current study, salt stress enhanced the MDA activity in rice plants (**Figure 4A**). Similar to our study, the observed increase in malondialdehyde (MDA) content is likely a consequence of an imbalance caused by the excessive accumulation of reactive oxygen species (ROS) within organelles such as mitochondria and peroxisomes (He et al., 2021). In their study, Waqas et al. (2019) reported that adding biochar to the soil can increase plant hormone production, which helps plants better defend themselves against different environmental challenges. The treatments activated the rice plants’ self-defense systems, which responded to the increased levels of ROS (**Figure 4**). Similarly, Hafeez et al. (2024) reported that adding biochar to the soil increased the activity of the enzymes CAT, APX, and POX, which helped to reduce the negative impacts of salt-contaminated soil. This protective system consists of a group of antioxidant enzymes, such as superoxide dismutase (SOD), peroxidase (POD), and catalase (CAT). These enzymes work together to remove and neutralize different ROS molecules, preventing cellular components from being damaged by oxidation (Caverzan et al., 2016). Increasing the activity of antioxidant enzymes helped to reduce oxidative stress caused by salt stress, which, in turn, decreased the levels of ROS (Raza et al., 2012; Abo-Elyousr et al., 2022; Zhang et al., 2019b). Additionally, numerous studies have shown that N fertilizer can be a primary defense against both internal and external oxidative stressors (Ahanger et al., 2019; Khan et al., 2017). Applying nitrogen fertilizer can increase the activity of SOD, POD, CAT, and free amino acids, which help to remove ROS molecules (Sikder et al., 2020). Many studies suggest that salt-tolerant plant varieties have higher levels of antioxidant enzymes and/or non-enzymatic antioxidants, which help them better tolerate salt (Moradi and Ismail, 2007; Gill and Tuteja, 2010; Wang et al., 2019).

When plants are under stress, increasing the levels of proline and soluble sugars can help them better tolerate it (Khan et al., 2019; Sami et al., 2016). In many studies, it was reported that proline and soluble sugars are compatible solutes that help maintain the osmotic balance in plants under salt stress (Khan et al., 2023; Munns and Tester, 2008). Moreover, these osmotic substances can help stabilize proteins and their complexes as well as the stability of cell membranes under salt stress by reducing the negative effects of high salt concentrations (Na^+^ and Cl^─^) on the activity of enzymes (Feghhenabi et al., 2020; Etesami et al., 2021). It has recently been found that combining the use of microbes (AMF) and biochar can significantly increase the proline content in plants (Yan et al., 2020). Moreover, the highest values of total soluble sugar (TSS) were reported in the PGPR + biochar treatment under saline water conditions (Nehela et al., 2021). Similarly, it was also reported that N improves soluble sugars under stressed conditions (Ikeyama et al., 2020). The combined application of biochar and inorganic fertilizers (NPK) increased the levels of proline and soluble sugars in plants under stressful conditions (El-Syed et al., 2023). Furthermore, according to Sikder et al. (2020), the ST genotype accumulated more osmoprotectants than Z0102, which helped them absorb water better and to be more tolerant to salt. Many studies reported high antioxidant enzyme, proline, and TSS contents in stress-tolerant cultivars compared to sensitive cultivars (Khan et al., 2019; Liu et al., 2016).

### 4.5 Microbial biochar with N maintain RWC, Ψw, and MSI under saline conditions

Under stress conditions, changes in membrane stability and water potential are considered as primary injury (Nandwal et al., 2019; Awaji et al., 2022). In the current study, it was observed that salt stress has a significant negative effect on RWC, water potential, and MSI (**Figure 5**). Our findings align with many other studies which have shown that under salt stress, a decrease in Ψw and RWC is caused by disruptions in the plant’s water content (Sikder et al., 2020; Soni et al., 2021). High soil salinity, primarily due to NaCl, presents a dual challenge for plants: osmotic and ionic stress and both stresses restrict the plant’s capacity to take up water and essential minerals, hindering its growth and development (Singh et al., 2018). According to Sikder et al. (2020), soil salinization causes osmotic stress, which can lead to physiological drought that is characterized by reduced water uptake by the roots, decreased internal water availability, and impaired water metabolism, transport, cooling, and other growth-related processes. However, osmotic regulation is an effective strategy that plants can use to adapt to dehydration (Hussain Wani et al., 2013; Singh et al., 2015). In this study, the combination of biochar BB + N120 increased the accumulation of compatible osmotic substances like proline and soluble sugar in plants exposed to salt stress (**Figures 4E, F**). Similar to our findings, Sikder et al. (2020) also reported that adequate N fertilizer application improved osmotic regulation, thereby ensuring sufficient water and mineral uptake for carbon and nitrogen metabolism. Moreover, biochar amendments are a promising approach to mitigating the harmful effects of osmotic stress on plants. One key mechanism involves biochar’s ability to enhance water retention within the soil matrix, thereby mitigating the water stress experienced by plants (Lyu et al., 2016). Furthermore, biochar application has been demonstrated to stimulate the production of cytokinins in the soil, which can enhance relative water content (RWC) within plant leaves under conditions of osmotic stress (Kumari et al., 2018; Murtaza et al., 2024). According to Ran et al. (2020), biochar application can preserve the integrity of rice cells by mitigating the damage to the cell membrane caused by sodium stress and osmotic stress, ultimately leading to increased leaf water content as biochar can reduce the sodium content and increase the potassium (an inorganic osmoprotectant) content in rice plant organs.

Plants exhibiting a higher membrane stability index (MSI) under stress conditions display a form of stress adaptation as evidenced by their ability to maintain membrane integrity (Bangar et al., 2019). This reinforcement is achieved by enhancing the activity of protective enzymes and facilitating electron transfer processes within the leaves. Salt-tolerant bacteria produced organic osmolytes, including highly soluble organic compounds 1-aminocyclopropane-1-carboxylate (ACC) deaminase, indole-3-acetic acid (IAA), antioxidants, extracellular polymeric substance (EPS), and volatile organic compounds (VOC) (Sikder et al., 2020). Some osmolytes are produced by microbes, and others are accumulated externally. In the current study, BB + N120 enhanced the abundance of salt-tolerant bacteria (**Figure 3C**) and also enhanced osmolytes in plants under salt stress (**Figures 4E, F**). Previously, in their studies, Suprasanna et al. (2016) also reported the same organic osmolytes that helped plants during osmotic stress. These findings highlight the potential of BB + N120 fertilization as a powerful strategy to mitigate the negative effects of salt stress on rice crops and improve their overall stress tolerance.

### 4.6 Microbial biochar combined with N enhanced the physiological performance of rice plants under salt stress

Salinity has negative effects on the photosynthetic process of plant and the production of the enzyme chlorophyllase (Elsaeed et al., 2021; Wang et al., 2025). Severe atmospheric conditions that are detrimental for photosynthetic equipment include salt stress (Fareed et al., 2024). However, studies by Wang et al. (2022); Jaiswal et al. (2020) suggest that biochar and N application can enhance plant photosynthesis and carbon fixation, potentially by mitigating chlorophyll degradation and promoting mineral homeostasis within the soil. Moreover, biochar enhances photosynthesis by stimulating chlorophyll synthesis, electron transport chain activity, and photosystem I and II function. According to Ghassemi-Golezani and Farhangi-Abriz (2019), applying biochar to the soil reduced the production of reactive oxygen species (ROS) and significantly increased the chlorophyll levels, ultimately leading to a higher maximum quantum yield efficiency of photosystem II. According to Altaf et al. (2024), it is inevitable for N to enhance the photosynthesis performance of plants. Application of nitrogen at appropriate rates has been shown to optimize photosynthetic processes, increase leaf area, and elevate net assimilation rates (Din et al., 2021). Based on our results, we can conclude that SLY138 is salt-resistant cultivar. Consistent with our findings, Khan et al. (2019) also reported that salt-resistant cultivars exhibited a smaller decline in photosynthetic attributes. Many previous studies also reported that stress-tolerant genotypes showed less reduction in photosynthesis, which indicated that photo-inhibition in PSII in tolerant genotypes was less (Zhang et al., 2015a, Zhang et al., 2015b).

### 4.7 Microbial-inoculated biochar with N improved the stomatal structure of rice plants under salt stress

Na^+^ and Cl^─^ accumulation in leaves can trigger stomatal closure and decrease the chlorophyll content, ultimately limiting photosynthesis (Kwon et al., 2019). However, Paneque et al. (2016) suggested that biochar application can mitigate the effects of stress on plant stomata, potentially due to biochar’s water conservation properties. Interestingly, biochar incorporation increased both stomatal aperture and density in plants, suggesting reduced stress (Akhtar et al., 2015). Plants treated with a combination of biochar and nitrogen exhibited superior stomatal traits compared to the control and single high-nitrogen treatments (Khan et al., 2021). There are two possible mechanisms adopted by biochar. First, according to Sial et al. (2019), its porous structure and high surface area facilitate moisture retention, leading to increased soil-water-holding capacity and greater water availability for root uptake, resulting in higher stomatal conductance. Second, biochar application has been shown to reduce stomatal injury by promoting soil water and nutrient availability as well as electron transport rates in photosystem II, ultimately enhancing plant growth (Haider et al., 2015).

### 4.8 Microbial biochar with N modulated K^+^/Na^+^ balance in rice plants under saline conditions

In our study, ionic imbalance was observed in plants under saline conditions (**Figures 7A–C**). Similarly to our findings, Parihar et al. (2015) reported that salinity enhances the accumulation of Na^+^ and decreased K^+^ in plants during its ionic stress mechanism. However, the efficiency of plants to adjust the Na^+^/K^+^ ratio is salt tolerance (Rameeh, 2012; Jayakannan et al., 2013). Biochar can be applied as a mitigation strategy in salt-affected soils to reduce salinity stress due to its capacity to adsorb sodium ions (Na^+^) onto its surface (Zhang et al., 2019). In the current study, the adsorption capacity of microbial biochars (BF and BB) is doubled due to bioadsorption. The study of Cui et al. (2021) also have similar results, demonstrating that the combined application of biochar and effective microorganisms was more effective in mitigating salinity effects than biochar alone. Furthermore, nitrogen application can decrease sodium (Na^+^) uptake in plants by stimulating the production of plasma-membrane-bound translocating proteins (Yao et al., 2021). Nitrogen application decreased the Na^+^ content and increased the K^+^ content by increasing the content of osmoregulatory substances and improving osmotic adjustment (Tian et al., 2022). In the current study, it is observed that BB + N120 enhanced more osmotic substances (proline and soluble sugars) compared to the BC treatment alone (**Figures 4E, F**). Our findings were consistent with those of Liu et al. (2020), who also reported variations in K^+^/Na^+^ content among different cultivars. Similarly, Singh et al. (2018) also observed that the salt-tolerant rice variety CSR10 maintained a lower Na^+^/K^+^ ratio in the shoots compared to the salt-sensitive variety MI 48 by restricting the translocation of sodium (Na^+^) from the roots to the shoots. Furthermore, to support our study, Zhang et al. (2019) also reported that Nipponbare, a salt-sensitive rice variety, absorbed higher levels of sodium (Na^+^) and chloride (Cl^─^) compared to Jinyuan 85.

### 4.9 Microbial biochar and N synergistically improved the agronomic characteristics and SPAD values of rice plants under salt stress

In the current study, agronomic characteristics such as plant height, fresh weight, dry weight, and the number of tillers, as well as SPAD values, were reduced under saline conditions (**Table 1**) due to disruption of Na^+^/K^+^ balance. According to Hussain et al. (2021), this imbalance damages plant leaves and the root system, negatively impacting the photosynthetic efficiency and, ultimately, growth. Moreover, soil physicochemical properties such as nutrient deficiencies, ionic toxicity, and osmotic stress are the main factors contributing to these diminished agronomic characteristics under saline stress (Morsy et al., 2022). However, biochar can substantially enhance the agronomic characteristics by improving soil physicochemical properties and nutrient availability, stimulating soil microbial activity, and optimizing root morphological and physiological traits (Cui et al., 2021; Joseph et al., 2021). In current study, we observed that BB + N120 significantly improved several rice growth parameters—plant height (PH), plant fresh weight (PFW), plant dry weight (PDW), number of tillers, and SPAD content (**Table 3**)—by different mechanisms. Firstly, BB + N120 improved the overall physicochemical properties of saline soil (**Figure 2**) Secondly, this treatment suppressed the MDA activity and increased the antioxidant activities and osmo-regulators (**Figure 4A**). Thirdly, by maintaining a favorable K^+^/Na^+^ ionic balance in leaves (**Figure 7C**). Similarly, when biochar combined with N is added in the paddy field, it improved various aspects of rice growth and yield (Gu et al., 2022; Sun et al., 2022). The combined application of biochar and nitrogen significantly enhanced further the plant height, tiller number, and leaf area than a single application of either biochar or nitrogen fertilizer, which might be due to the high amount and increased availability of nutrients (Khan et al., 2021). Generally, N greatly influences the aboveground biomass of plants by promoting tillering before stem extension (Abbruzzini et al., 2019; Ullah et al., 2021). In many studies, it was reported that biochar and N are both more favorable for soil microorganisms and soil enzyme activities, improving root exudates and optimizing plant growth (Jin et al., 2024; Zhang et al., 2024). Since using microbial-modified biochar (BF and BB) with nitrogen fertilizer seems so beneficial, more studies are needed to understand the synergistic effect of microbial biochar and chemical fertilizers (N, P, and K) and also to optimize the dose of these fertilizers and modified biochar, making them more effective in saline soils. In our study, the efficiency of our selected treatments was in this order: BC < BF < BB < BF + N < BB + N. Our study clearly shows that adding 1% microbial biochar with N at the rate of 120 kg/ha to very salty rice fields can be a powerful way to boost rice production.

The correlation analysis (**Figures 8A, B**) revealed strong and consistent relationships among soil properties, ionic balance, physiological traits, and rice growth under saline conditions. Growth attributes (PFW, PDW, plant height, tiller number, and SPAD) were positively correlated with K^+^ concentration, K^+^/Na^+^ ratio, osmolytes (proline and total soluble sugars), and antioxidant enzyme activities (SOD, POD, and CAT), indicating that improved ionic homeostasis and oxidative stress mitigation were key drivers of biomass accumulation. In contrast, Na^+^ concentration and MDA showed strong negative correlations with growth and physiological traits, highlighting the detrimental effects of ionic toxicity and lipid peroxidation under salinity stress. Soil organic matter, NH_4_^+^–N, and microbial biomass were positively associated with antioxidant activity and plant water status, suggesting that microbial-inoculated biochar enhanced the nutrient availability and stress resilience through improved soil biochemical functioning. Overall, the correlation patterns confirm that the synergistic application of microbial biochar and nitrogen alleviated salinity stress by coordinating soil improvement, Na^+^ exclusion, antioxidant defense, and osmotic adjustment, ultimately promoting rice growth.

## 5 Conclusions

Plant growth is strongly constrained under saline stress; however, amending saline paddy soil with microbial-inoculated biochar (BB) combined with nitrogen markedly improved the soil chemical properties, particularly organic matter and available nitrogen (NH_4_^+^–N), thereby supporting rice nutrient demand throughout the growth period compared with the application of simple biochar alone. Microbial-modified biochar treatments enhanced the microbial abundance and diversity, especially pollution-degrading *Desulfobacterota*, salt-tolerant *Actinobacterota*, and organic-matter-decomposing *Ascomycota*, which collectively contributed to salt ion regulation, improved nitrification processes, and accelerated organic matter turnover.

At the plant level, BB combined with nitrogen (BB + N120) effectively suppressed reactive oxygen species accumulation and enhanced antioxidant enzyme activities, osmolyte production, leaf water potential, and relative water content compared with the BC treatment. These coordinated soil–microbe–plant responses demonstrate that microbial-modified biochar combined with nitrogen improves rice growth under saline conditions by simultaneously enhancing soil fertility, microbial functionality, and plant self-defense mechanisms.

From an applied perspective, these findings highlight the potential of microbial-inoculated biochar–nitrogen combinations as a practical soil amendment for saline and saline–alkaline paddy fields. Future field-scale studies are needed to evaluate the long-term stability of microbial colonization, nutrient dynamics, and biochar persistence as well as to assess the effectiveness of this strategy across different rice genotypes and other salt-sensitive crops. Such efforts will be critical to translate this approach into sustainable agricultural management practices under salinity stress.

## Data Availability

The raw sequence data reported in this paper have been deposited in the Genome Sequence Archive (GSA) at the National Genomics Data Center, China National Center for Bioinformation, under accession number CRA039853, and are publicly accessible at https://ngdc.cncb.ac.cn/gsa.

## References

[B1] Abbas H. M. M. Rais U. Altaf M. M. Rasul F. Shah A. Tahir A. . (2024a). Microbial-inoculated biochar for remediation of salt and heavy metal contaminated soils. Sci. Tot. Environ. 954, 176104. doi: 10.1016/j.scitotenv.2024.176104, PMID: 39250966

[B2] Abbas H. M. M. Rais U. Sultan H. Tahir A. Bahadur S. Shah A. . (2024b). Residual effect of microbial-inoculated biochar with nitrogen on rice growth and salinity reduction in paddy soil. Plants 13, 2804. doi: 10.3390/plants13192804, PMID: 39409674 PMC11478880

[B3] Abbruzzini T. F. Davies C. A. Toledo F. H. Cerri C. E. P. (2019). Dynamic biochar effects on nitrogen use efficiency, crop yield and soil nitrous oxide emissions during a tropical wheat-growing season. J. Environ. Manage. 252, 109638. doi: 10.1016/j.jenvman.2019.109638, PMID: 31586743

[B4] Abo-Elyousr K. A. M. Mousa M. A. A. Ibrahim O. H. M. Alshareef N. O. Eissa M. A. (2022). Calcium-rich biochar stimulates salt resistance in pearl millet (Pennisetum glaucum L.) plants by improving soil quality and enhancing the antioxidant defense. Plants 11, 1301. doi: 10.3390/plants11101301, PMID: 35631726 PMC9145951

[B5] Aebi H. (1984). “ [13] catalase *in vitro*,” in Methods in enzymology ( Elsevier), 121–126. doi: 10.1016/S0076-6879(84)05016-3, PMID: 6727660

[B6] Ahanger M. A. Qin C. Begum N. Maodong Q. Dong X. X. El-Esawi M. . (2019). Nitrogen availability prevents oxidative effects of salinity on wheat growth and photosynthesis by up-regulating the antioxidants and osmolytes metabolism, and secondary metabolite accumulation. BMC Plant Biol. 19, 1–12. doi: 10.1186/s12870-019-2085-3, PMID: 31703619 PMC6839093

[B7] Ajibade F. O. Yin W.-X. Guadie A. Ajibade T. F. Liu Y. Kumwimba M. N. . (2023). Impact of biochar amendment on antibiotic removal and ARGs accumulation in constructed wetlands for low C/N wastewater treatment. Chem. Eng. J. 459, 141541. doi: 10.1016/j.cej.2023.141541, PMID: 41853590

[B8] Akhtar S. S. Andersen M. N. Liu F. (2015). Biochar mitigates salinity stress in potato. J. Agron. Crop Sci. 201, 368–378. doi: 10.1111/jac.12132, PMID: 41834780

[B9] Altaf A. Quan M. Aleem M. Iqbal Z. Zhu M. Zhu X. (2024). Study of the effects of different environmental stresses on grain yield and associated traits of wheat grown with controlled release nitrogen fertilizer (CRNF). Pakistan J. Agric. Sci. 61, 337–350. Available online at: http://www.pakjas.com.pk

[B10] Ameloot N. Graber E. R. Verheijen F. G. A. De Neve S. (2013). Interactions between biochar stability and soil organisms: review and research needs. Eur. J. Soil Sci. 64, 379–390. doi: 10.1111/ejss.12064, PMID: 41834780

[B11] Amundson R. Berhe A. A. Hopmans J. W. Olson C. Sztein A. E. Sparks D. L. (2015). Soil and human security in the 21st century. Sci. (80-.). 348, 1261071. doi: 10.1126/science.1261071, PMID: 25954014

[B12] Awaji S. M. Hanjagi P. S. Repudi S. R. Suravi U. S. Baig M. J. Swain P. (2022). Identification and characterization of drought tolerant rice genotypes using physiological and biochemical traits. Oryza. 221–231. doi: 10.35709/ory.2022.59.2.12

[B13] Bangar P. Chaudhury A. Tiwari B. Kumar S. Kumari R. Bhat K. V. (2019). Morphophysiological and biochemical response of mungbean [Vigna radiata (L.) Wilczek] varieties at different developmental stages under drought stress. Turkish. J. Biol. 43, 58–69. doi: 10.3906/biy-1801-64, PMID: 30930636 PMC6426646

[B14] Barrs H. D. Weatherley P. E. (1962). A re-examination of the relative turgidity technique for estimating water deficits in leaves. Aust. J. Biol. Sci. 15, 413–428. doi: 10.1071/BI9620413, PMID: 41161682

[B15] Bates L. S. Waldren R. P. A. Teare I. D. (1973). Rapid determination of free proline for water-stress studies. Plant Soil 39, 205–207. doi: 10.1007/BF00018060, PMID: 41853694

[B16] Bello A. Wang B. Zhao Y. Yang W. Ogundeji A. Deng L. . (2021). Composted biochar affects structural dynamics, function and co-occurrence network patterns of fungi community. Sci. Tot. Environ. 775, 145672. doi: 10.1016/j.scitotenv.2021.145672, PMID: 33618307

[B17] Caverzan A. Casassola A. Brammer S. P. (2016). Reactive oxygen species and antioxidant enzymes involved in plant tolerance to stress. Abiotic Biot. Stress Plants-recent. Adv. Futur. Perspect. 17, 463–480. doi: 10.5772/61368

[B18] Chance B. Maehly A. C. (1955). Assay of catalases and peroxidases: methods in enzymology. Acad. Press. 2, 764–775. doi: 10.1016/S0076-6879(55)02300-8, PMID: 41578029

[B19] Chen C. R. Phillips I. R. Condron L. M. Goloran J. Xu Z. H. Chan K. Y. (2013). Impacts of greenwaste biochar on ammonia volatilisation from bauxite processing residue sand. Plant Soil 367, 301–312. doi: 10.1007/s11104-012-1468-0, PMID: 41853694

[B20] Cheng Z. Chen Y. Zhang F. (2018). Effect of reclamation of abandoned salinized farmland on soil bacterial communities in arid northwest China. Sci. Tot. Environ. 630, 799–808. doi: 10.1016/j.scitotenv.2018.02.259, PMID: 29494981

[B21] Cui Q. Xia J. Yang H. Liu J. Shao P. (2021). Biochar and effective microorganisms promote Sesbania cannabina growth and soil quality in the coastal saline-alkali soil of the Yellow River Delta, China. Sci. Tot. Environ. 756, 143801. doi: 10.1016/j.scitotenv.2020.143801, PMID: 33307496

[B22] Dahlawi S. Naeem A. Rengel Z. Naidu R. (2018). Biochar application for the remediation of salt-affected soils: Challenges and opportunities. Sci. Tot. Environ. 625, 320–335. doi: 10.1016/j.scitotenv.2017.12.257, PMID: 29289780

[B23] Dendooven L. Alcántara-Hernández R. J. Valenzuela-Encinas C. Luna-Guido M. Perez-Guevara F. Marsch R. (2010). Dynamics of carbon and nitrogen in an extreme alkaline saline soil: a review. Soil Biol. Biochem. 42, 865–877. doi: 10.1016/j.soilbio.2010.02.014, PMID: 41853590

[B24] Dhal B. Thatoi H. Das N. Pandey B. D. (2010). Reduction of hexavalent chromium by Bacillus sp. isolated from chromite mine soils and characterization of reduced product. J. Chem. Technol. Biotechnol. 85, 1471–1479. doi: 10.1002/jctb.2451, PMID: 41848424

[B25] Din I. Khan H. Khan N. A. Khil A. (2021). Inoculation of nitrogen fixing bacteria in conjugation with integrated nitrogen sources induced changes in phenology, growth, nitrogen assimilation and productivity of wheat crop. J. Saudi. Soc Agric. Sci. 20, 459–466. doi: 10.1016/j.jssas.2021.05.008, PMID: 41853590

[B26] El-Naggar A. Lee S. S. Rinklebe J. Farooq M. Song H. Sarmah A. K. . (2019). Biochar application to low fertility soils: A review of current status, and future prospects. Geoderma 337, 536–554. doi: 10.1016/j.geoderma.2018.09.034, PMID: 41853590

[B27] Elsaeed S. M. Zaki E. G. Ibrahim T. M. Ibrahim Talha N. Saad H. A. Gobouri A. A. . (2021). Biochar grafted on CMC-terpolymer by green microwave route for sustainable agriculture. Agriculture 11, 350. doi: 10.3390/agriculture11040350, PMID: 41725453

[B28] El-Sayed M. E. A. Hazman M. Abd El-Rady A. G. Almas L. McFarland M. Shams El Din A. . (2021). Biochar reduces the adverse effect of saline water on soil properties and wheat production profitability. Agric. 11, 1112. doi: 10.3390/agriculture11111112, PMID: 41725453

[B29] El-Syed N. M. M. Helmy A. M. Fouda S. E. E. Nabil M. M. Abdullah T. A. Alhag S. K. . (2023). Biochar with organic and inorganic fertilizers improves defenses, nitrogen use efficiency, and yield of maize plants subjected to water deficit in an alkaline soil. Sustainability 15, 12223. doi: 10.3390/su151612223, PMID: 41725453

[B30] Etesami H. Fatemi H. Rizwan M. (2021). Interactions of nanoparticles and salinity stress at physiological, biochemical and molecular levels in plants: A review. Ecotoxicol. Environ. Saf. 225, 112769. doi: 10.1016/j.ecoenv.2021.112769, PMID: 34509968

[B31] Fareed S. Haider A. Ramzan T. Ahmad M. Younis A. Zulfiqar U. . (2024). Investigating the growth promotion potential of biochar on pea (Pisum sativum) plants under saline conditions. Sci. Rep. 14, 10870. doi: 10.1038/s41598-024-59891-x, PMID: 38740776 PMC11091058

[B32] Fedeli R. Vannini A. Djatouf N. Celletti S. Loppi S. (2024). Can lettuce plants grow in saline soils supplemented with biochar? Heliyon 10. doi: 10.1016/j.heliyon.2024.e26526, PMID: 38404867 PMC10884517

[B33] Feghhenabi F. Hadi H. Khodaverdiloo H. Van Genuchten M. T. (2020). Seed priming alleviated salinity stress during germination and emergence of wheat (Triticum aestivum L.). Agric. Water Manage. 231, 106022. doi: 10.1016/j.agwat.2020.106022, PMID: 41853590

[B34] Ghassemi-Golezani K. Farhangi-Abriz S. (2019). Biochar alleviates fluoride toxicity and oxidative stress in safflower (Carthamus tinctorius L.) seedlings. Chemosphere 223, 406–415. doi: 10.1016/j.chemosphere.2019.02.087, PMID: 30784747

[B35] Gill S. S. Tuteja N. (2010). Reactive oxygen species and antioxidant machinery in abiotic stress tolerance in crop plants. Plant Physiol. Biochem. 48, 909–930. doi: 10.1016/j.plaphy.2010.08.016, PMID: 20870416

[B36] Gu W. Wang Y. Feng Z. Wu D. Zhang H. Yuan H. . (2022). Long-term effects of biochar application with reduced chemical fertilizer on paddy soil properties and japonica rice production system. Front. Environ. Sci. 10, 902752. doi: 10.3389/fenvs.2022.902752, PMID: 41853828

[B37] Guo T. Zhang G. Zhou M. Wu F. Chen J. (2004). Effects of aluminum and cadmium toxicity on growth and antioxidant enzyme activities of two barley genotypes with different Al resistance. Plant Soil 258, 241–248. doi: 10.1023/B:PLSO.0000016554.87519.d6, PMID: 38124636

[B38] Hafeez M. B. Ghaffar A. Zahra N. Ahmad N. Hussain S. Li J. (2024). Plant growth promoters boost the photosynthesis related mechanisms and secondary metabolism of late-sown wheat under contrasting saline regimes. Plant Stress 12, 100480. doi: 10.1016/j.stress.2024.100480, PMID: 41853590

[B39] Haider G. Koyro H.-W. Azam F. Steffens D. Müller C. Kammann C. (2015). Biochar but not humic acid product amendment affected maize yields via improving plant-soil moisture relations. Plant Soil 395, 141–157. doi: 10.1007/s11104-014-2294-3, PMID: 41853694

[B40] He W. Yan K. Zhang Y. Bian L. Mei H. Han G. (2021). Contrasting photosynthesis, photoinhibition and oxidative damage in honeysuckle (Lonicera japonica Thunb.) under iso-osmotic salt and drought stresses. Environ. Exp. Bot. 182, 104313. doi: 10.1016/j.envexpbot.2020.104313, PMID: 41853590

[B41] He L. Zhong H. Liu G. Dai Z. Brookes P. C. Xu J. (2019). Remediation of heavy metal contaminated soils by biochar: Mechanisms, potential risks and applications in China. Environ. pollut. 252, 846–855. doi: 10.1016/j.envpol.2019.05.151, PMID: 31202137

[B42] Heath R. L. Packer L. (1968). Photoperoxidation in isolated chloroplasts: I. Kinetics and stoichiometry of fatty acid peroxidation. Arch. Biochem. Biophys. 125, 189–198. doi: 10.1016/0003-9861(68)90654-1, PMID: 5655425

[B43] Hou Q. Zuo T. Wang J. Huang S. Wang X. Yao L. . (2021). Responses of nitrification and bacterial community in three size aggregates of paddy soil to both of initial fertility and biochar addition. Appl. Soil Ecol. 166, 104004. doi: 10.1016/j.apsoil.2021.104004, PMID: 41853590

[B44] Huang S. Huang P. Hareem M. Tahzeeb-ul-Hassan M. Younis U. Dawar K. . (2024). Evaluating the hidden potential of deashed biochar in mitigating salinity stress for cultivation of fenugreek. Sci. Rep. 14, 141. doi: 10.1038/s41598-023-49063-8, PMID: 38167554 PMC10761952

[B45] Huang J. Xiao Y. Chen B. (2023). Nutrients removal by Olivibacter jilunii immobilized on activated carbon for aquaculture wastewater treatment: ppk1 gene and bacterial community structure. Bioresour. Technol. 370, 128494. doi: 10.1016/j.biortech.2022.128494, PMID: 36526116

[B46] Hussain S. Hussain S. Ali B. Ren X. Chen X. Li Q. . (2021). Recent progress in understanding salinity tolerance in plants: Story of Na+/K+ balance and beyond. Plant Physiol. Biochem. 160, 239–256. doi: 10.1016/j.plaphy.2021.01.029, PMID: 33524921

[B47] Hussain Wani S. Brajendra Singh N. Haribhushan A. Iqbal Mir J. (2013). Compatible solute engineering in plants for abiotic stress tolerance-role of glycine betaine. Curr. Genomics 14, 157–165. doi: 10.2174/1389202911314030001, PMID: 24179438 PMC3664465

[B48] Hussein M. A. A. Alqahtani M. M. Alwutayd K. M. Aloufi A. S. Osama O. Azab E. S. . (2023). Exploring salinity tolerance mechanisms in diverse wheat genotypes using physiological, anatomical, agronomic and gene expression analyses. Plants 12, 3330. doi: 10.3390/plants12183330, PMID: 37765494 PMC10535590

[B49] Ikeyama N. Ohkuma M. Sakamoto M. (2020). Stress response of Mesosutterella multiformis mediated by nitrate reduction. Microorganisms 8, 2003. doi: 10.3390/microorganisms8122003, PMID: 33333944 PMC7765368

[B50] Jaiswal A. K. Alkan N. Elad Y. Sela N. Graber E. R. Frenkel O. (2020). Molecular insights into biochar-mediated plant growth promotion and systemic resistance in tomato against Fusarium crown and root rot disease. Sci. Rep. 10, 13934. doi: 10.1038/s41598-020-70882-6, PMID: 32811849 PMC7434890

[B51] Jameel J. Anwar T. Majeed S. Qureshi H. Siddiqi E. H. Sana S. . (2024). Effect of salinity on growth and biochemical responses of brinjal varieties: implications for salt tolerance and antioxidant mechanisms. BMC Plant Biol. 24, 128. doi: 10.1186/s12870-024-04836-9, PMID: 38383291 PMC10880304

[B52] Jayakannan M. Bose J. Babourina O. Rengel Z. Shabala S. (2013). Salicylic acid improves salinity tolerance in Arabidopsis by restoring membrane potential and preventing salt-induced K+ loss via a GORK channel. J. Exp. Bot. 64, 2255–2268. doi: 10.1093/jxb/ert085, PMID: 23580750 PMC3654417

[B53] Jin F. Piao J. Miao S. Che W. Li X. Li X. . (2024). Long-term effects of biochar one-off application on soil physicochemical properties, salt concentration, nutrient availability, enzyme activity, and rice yield of highly saline-alkali paddy soils: based on a 6-year field experiment. Biochar 6, 1–22. doi: 10.1007/s42773-024-00332-3, PMID: 41853694

[B54] Joseph S. Cowie A. L. Van Zwieten L. Bolan N. Budai A. Buss W. . (2021). How biochar works, and when it doesn’t: A review of mechanisms controlling soil and plant responses to biochar. Gcb. Bioenergy 13, 1731–1764. doi: 10.1111/gcbb.12885, PMID: 41834780

[B55] Karlen D. L. Rice C. W. (2015). Soil degradation: Will humankind ever learn? Sustainability 7, 12490–12501. doi: 10.3390/su70912490, PMID: 41725453

[B56] Karthik C. Barathi S. Pugazhendhi A. Ramkumar V. S. Thi N. B. D. Arulselvi P. I. (2017). Evaluation of Cr (VI) reduction mechanism and removal by Cellulosimicrobium funkei strain AR8, a novel haloalkaliphilic bacterium. J. Hazard. Mater. 333, 42–53. doi: 10.1016/j.jhazmat.2017.03.037, PMID: 28340388

[B57] Khan M. N. Fu C. Li J. Tao Y. Li Y. Hu J. . (2023). Seed nanopriming: How do nanomaterials improve seed tolerance to salinity and drought? Chemosphere 310, 136911. doi: 10.1016/j.chemosphere.2022.136911, PMID: 36270526

[B58] Khan M. N. Fu C. Liu X. Li Y. Yan J. Yue L. . (2024). Nanopriming with selenium doped carbon dots improved rapeseed germination and seedling salt tolerance. Crop J. 12, 1333–1343. doi: 10.1016/j.cj.2024.03.007, PMID: 41853590

[B59] Khan Z. Nauman Khan M. Luo T. Zhang K. Zhu K. Rana M. S. . (2021). Compensation of high nitrogen toxicity and nitrogen deficiency with biochar amendment through enhancement of soil fertility and nitrogen use efficiency promoted rice growth and yield. Gcb. Bioenergy 13, 1765–1784. doi: 10.1111/gcbb.12884, PMID: 41834780

[B60] Khan A. Tan D. K. Y. Afridi M. Z. Luo H. Tung S. A. Ajab M. . (2017). Nitrogen fertility and abiotic stresses management in cotton crop: a review. Environ. Sci. pollut. Res. 24, 14551–14566. doi: 10.1007/s11356-017-8920-x, PMID: 28434155

[B61] Khan M. N. Zhang J. Luo T. Liu J. Ni F. Rizwan M. . (2019). Morpho-physiological and biochemical responses of tolerant and sensitive rapeseed cultivars to drought stress during early seedling growth stage. Acta Physiol. Plant 41, 1–13. doi: 10.1007/s11738-019-2812-2, PMID: 41853694

[B62] Kong Y. Xu X. Zhu L. (2013). Cyanobactericidal effect of Streptomyces sp. HJC-D1 on Microcystis auruginosa. PloS One 8, e57654. doi: 10.1371/journal.pone.0057654, PMID: 23460891 PMC3584028

[B63] Kono Y. (1978). Generation of superoxide radical during autoxidation of hydroxylamine and an assay for superoxide dismutase. Arch. Biochem. Biophys. 186, 189–195. doi: 10.1016/0003-9861(78)90479-4, PMID: 24422

[B64] Kumari S. Kumar S. Prakash P. (2018). Exogenous application of cytokinin (6-BAP) ameliorates the adverse effect of combined drought and high temperature stress in wheat seedling. J. Pharmacogn. Phytochem. 7, 1176–1180. Available online at: https://www.phytojournal.com

[B65] Kwon O. K. Mekapogu M. Kim K. S. (2019). Effect of salinity stress on photosynthesis and related physiological responses in carnation (Dianthus caryophyllus). Hortic. Environ. Biotechnol. 60, 831–839. doi: 10.1007/s13580-019-00189-7, PMID: 41853694

[B66] Lehmann J. Rillig M. C. Thies J. Masiello C. A. Hockaday W. C. Crowley D. (2011). Biochar effects on soil biota–a review. Soil Biol. Biochem. 43, 1812–1836. doi: 10.1016/j.soilbio.2011.04.022, PMID: 41853590

[B67] Li Y. L. Stanghellini C. (2001). Analysis of the effect of EC and potential transpiration on vegetative growth of tomato. Sci. Hortic. (Amsterdam). 89, 9–21. doi: 10.1016/S0304-4238(00)00219-3, PMID: 41334505

[B68] Li Z. Xing B. Ding Y. Li Y. Wang S. (2020). A high-performance biochar produced from bamboo pyrolysis with *in-situ* nitrogen doping and activation for adsorption of phenol and methylene blue. Chin. J. Chem. Eng. 28, 2872–2880. doi: 10.1016/j.cjche.2020.03.031, PMID: 41853590

[B69] Lian F. Xing B. (2017). Black carbon (biochar) in water/soil environments: molecular structure, sorption, stability, and potential risk. Environ. Sci. Technol. 51, 13517–13532. doi: 10.1021/acs.est.7b02528, PMID: 29116778

[B70] Liu D. Hu L. Y. Ali B. Yang A. G. Wan G. L. Xu L. . (2016). Influence of 5-aminolevulinic acid on photosynthetically related parameters and gene expression in Brassica napus L. under drought stress. Soil Sci. Plant Nutr. 62, 254–262. doi: 10.1080/00380768.2016.1198216, PMID: 41799851

[B71] Liu H. Xie Y. Li J. Zeng G. Li H. Xu F. . (2020). Effect of Serratia sp. K3 combined with organic materials on cadmium migration in soil-vetiveria zizanioides L. system and bacterial community in contaminated soil. Chemosphere 242, 125164. doi: 10.1016/j.chemosphere.2019.125164, PMID: 31669989

[B72] Lu W. Ding W. Zhang J. Zhang H. Luo J. Bolan N. (2015). Nitrogen amendment stimulated decomposition of maize straw-derived biochar in a sandy loam soil: a short-term study. PloS One 10, e0133131. doi: 10.1145/2818302, PMID: 26192282 PMC4507981

[B73] Luo Q. Hou D. Jiang D. Chen W. (2021). Bioremediation of marine oil spills by immobilized oil-degrading bacteria and nutrition emulsion. Biodegradation 32, 165–177. doi: 10.1007/s10532-021-09930-5, PMID: 33683578

[B74] Lyu S. Du G. Liu Z. Zhao L. Lyu D. (2016). Effects of biochar on photosystem function and activities of protective enzymes in Pyrus ussuriensis Maxim. under drought stress. Acta Physiol. Plant 38, 1–10. doi: 10.1007/s11738-016-2236-1, PMID: 41853694

[B75] Mehdizadeh L. Moghaddam M. Lakzian A. (2020). Amelioration of soil properties, growth and leaf mineral elements of summer savory under salt stress and biochar application in alkaline soil. Sci. Hortic. (Amsterdam). 267, 109319. doi: 10.1016/j.scienta.2020.109319, PMID: 41853590

[B76] Moradi F. Ismail A. M. (2007). Responses of photosynthesis, chlorophyll fluorescence and ROS-scavenging systems to salt stress during seedling and reproductive stages in rice. Ann. Bot. 99, 1161–1173. doi: 10.1093/aob/mcm052, PMID: 17428832 PMC3243573

[B77] Morsy S. Elbasyoni I. S. Baenziger S. Abdallah A. M. (2022). Gypsum amendment influences performance and mineral absorption in wheat cultivars grown in normal and saline-sodic soils. J. Agron. Crop Sci. 208, 675–692. doi: 10.1111/jac.12598, PMID: 41834780

[B78] Munns R. Tester M. (2008). Mechanisms of salinity tolerance. Annu. Rev. Plant Biol. 59, 651–681. doi: 10.1146/annurev.arplant.59.032607.092911, PMID: 18444910

[B79] Murtaza G. Usman M. Ahmed Z. Hyder S. Alwahibi M. S. Rizwana H. . (2024). Improving wheat physio-biochemical attributes in ciprofloxacin-polluted saline soil using nZVI-modified biochar. Ecotoxicol. Environ. Saf. 286, 117202. doi: 10.1016/j.ecoenv.2024.117202, PMID: 39490103

[B80] Nandwal A. S. Chand M. Singh K. Mishra A. K. Kumar A. Kumari A. . (2019). Varietal variation in physiological and biochemical attributes of sugarcane varieties under different soil moisture regimes. Indian J. Exp. Biol. 57, 721.

[B81] Nations U. (2022). Global issues: Population. Available online at: https://www.un.org/en/global-issues/population.

[B82] Nehela Y. Mazrou Y. S. A. Alshaal T. Rady A. M. S. El-Sherif A. M. A. Omara A. E.-D. . (2021). The integrated amendment of sodic-saline soils using biochar and plant growth-promoting rhizobacteria enhances maize (Zea mays L.) resilience to water salinity. Plants 10, 1960. doi: 10.3390/plants10091960, PMID: 34579492 PMC8466265

[B83] Novak J. M. Busscher W. J. Watts D. W. Laird D. A. Ahmedna M. A. Niandou M. A. S. (2010). Short-term CO2 mineralization after additions of biochar and switchgrass to a Typic Kandiudult. Geoderma 154, 281–288. doi: 10.1016/j.geoderma.2009.10.014, PMID: 41853590

[B84] Pan Y. Borjigin S. Liu Y. Wang H. Wang Y. Wu Y. . (2022). Role of key-stone microbial taxa in algae amended soil for mediating nitrogen transformation. Sci. Tot. Environ. 823, 153547. doi: 10.1016/j.scitotenv.2022.153547, PMID: 35101510

[B85] Paneque M. José M. Franco-Navarro J. D. Colmenero-Flores J. M. Knicker H. (2016). Effect of biochar amendment on morphology, productivity and water relations of sunflower plants under non-irrigation conditions. Catena 147, 280–287. doi: 10.1016/j.catena.2016.07.037, PMID: 41853590

[B86] Parihar P. Singh S. Singh R. Singh V. P. Prasad S. M. (2015). Effect of salinity stress on plants and its tolerance strategies: a review. Environ. Sci. pollut. Res. 22, 4056–4075. doi: 10.1007/s11356-014-3739-1, PMID: 25398215

[B87] Qadir M. Quillérou E. Nangia V. Murtaza G. Singh M. Thomas R. J. . (2014). “ Economics of salt-induced land degradation and restoration,” in Natural resources forum ( Wiley Online Library), 282–295. doi: 10.1111/1477-8947.12054, PMID:

[B88] Qi X. Gou J. Chen X. Xiao S. Ali I. Shang R. . (2021). Application of mixed bacteria-loaded biochar to enhance uranium and cadmium immobilization in a co-contaminated soil. J. Hazard. Mater. 401, 123823. doi: 10.1016/j.jhazmat.2020.123823, PMID: 33113745

[B89] Qi X. Xiao S. Chen X. Ali I. Gou J. Wang D. . (2022). Biochar-based microbial agent reduces U and Cd accumulation in vegetables and improves rhizosphere microecology. J. Hazard. Mater. 436, 129147. doi: 10.1016/j.jhazmat.2022.129147, PMID: 35643000

[B90] Qin F. Peng Y. Song G. Fang Q. Wang R. Zhang C. . (2020). Degradation of sulfamethazine by biochar-supported bimetallic oxide/persulfate system in natural water: Performance and reaction mechanism. J. Hazard. Mater. 398, 122816. doi: 10.1016/j.jhazmat.2020.122816, PMID: 32768858

[B91] Rameeh V. (2012). Ions uptake, yield and yield attributes of rapeseed exposed to salinity stress. J. Soil Sci. Plant Nutr. 12, 851–861. doi: 10.4067/S0718-95162012005000037, PMID: 27315006

[B92] Ran C. Gulaqa A. Zhu J. Wang X. Zhang S. Geng Y. . (2020). Benefits of biochar for improving ion contents, cell membrane permeability, leaf water status and yield of rice under saline–sodic paddy field condition. J. Plant Growth Regul. 39, 370–377. doi: 10.1007/s00344-019-09988-9, PMID: 41853694

[B93] Rath K. M. Fierer N. Murphy D. V. Rousk J. (2019). Linking bacterial community composition to soil salinity along environmental gradients. ISME. J. 13, 836–846. doi: 10.1038/s41396-018-0313-8, PMID: 30446737 PMC6461869

[B94] Raza M. A. S. Saleem M. F. Ashraf M. Y. Ali A. Asghar H. N. (2012). Glycinebetaine applied under drought improved the physiological efficiency of wheat (Triticum aestivum L.) plant. Soil Env. 31, 67–71. Available online at: http://www.se.org.pk

[B95] Reddy I. N. B. L. Kim B.-K. Yoon I.-S. Kim K.-H. Kwon T.-R. (2017). Salt tolerance in rice: focus on mechanisms and approaches. Rice Sci. 24, 123–144. doi: 10.1016/j.rsci.2016.09.004, PMID: 41853590

[B96] Sairam R. K. Deshmukh P. S. Shukla D. S. (1997). Tolerance of drought and temperature stress in relation to increased antioxidant enzyme activity in wheat. J. Agron. Crop Sci. 178, 171–178. doi: 10.1111/j.1439-037X.1997.tb00486.x, PMID: 41834780

[B97] Samaddar S. Truu J. Chatterjee P. Truu M. Kim K. Kim S. . (2019). Long-term silicate fertilization increases the abundance of Actinobacterial population in paddy soils. Biol. Fertil. Soil. 55, 109–120. doi: 10.1007/s00374-018-01335-6, PMID: 41853694

[B98] Sami F. Yusuf M. Faizan M. Faraz A. Hayat S. (2016). Role of sugars under abiotic stress. Plant Physiol. Biochem. 109, 54–61. doi: 10.1016/j.plaphy.2016.09.005, PMID: 27639065

[B99] Shah F. Nicolás C. Bentzer J. Ellström M. Smits M. Rineau F. . (2016). Ectomycorrhizal fungi decompose soil organic matter using oxidative mechanisms adapted from saprotrophic ancestors. New Phytol. 209, 1705–1719. doi: 10.1111/nph.13722, PMID: 26527297 PMC5061094

[B100] Sharma P. Tripathi A. Pandey M. (2022). A review: effects of nitrogenous fertilizers on soil (Ph, microbial community, greenhouse gases emission and carbon pool). Environ. Contam. Rev. 5, 44–48. doi: 10.26480/ecr.02.2022.44.48

[B101] Shi X. Hu H.-W. Wang J. He J.-Z. Zheng C. Wan X. . (2018). Niche separation of comammox Nitrospira and canonical ammonia oxidizers in an acidic subtropical forest soil under long-term nitrogen deposition. Soil Biol. Biochem. 126, 114–122. doi: 10.1016/j.soilbio.2018.09.004, PMID: 41853590

[B102] Sial T. A. Lan Z. Wang L. Zhao Y. Zhang J. Kumbhar F. . (2019). Effects of different biochars on wheat growth parameters, yield and soil fertility status in a silty clay loam soil. Molecules 24, 1798. doi: 10.3390/molecules24091798, PMID: 31075937 PMC6540089

[B103] Sikder R. K. Wang X. Zhang H. Gui H. Dong Q. Jin D. . (2020). Nitrogen enhances salt tolerance by modulating the antioxidant defense system and osmoregulation substance content in Gossypium hirsutum. Plants 9, 450. doi: 10.3390/plants9040450, PMID: 32260233 PMC7238023

[B104] Singh M. Kumar J. Singh S. Singh V. P. Prasad S. M. (2015). Roles of osmoprotectants in improving salinity and drought tolerance in plants: a review. Rev. Environ. Sci. Bio/Technol. 14, 407–426. doi: 10.1007/s11157-015-9372-8, PMID: 41853694

[B105] Singh J. Singh V. Sharma P. C. (2018). Elucidating the role of osmotic, ionic and major salt responsive transcript components towards salinity tolerance in contrasting chickpea (Cicer arietinum L.) genotypes. Physiol. Mol. Biol. Plants 24, 441–453. doi: 10.1007/s12298-018-0517-4, PMID: 29692552 PMC5911262

[B106] Soni S. Kumar A. Sehrawat N. Kumar A. Kumar N. Lata C. . (2021). Effect of saline irrigation on plant water traits, photosynthesis and ionic balance in durum wheat genotypes. Saudi. J. Biol. Sci. 28, 2510–2517. doi: 10.1016/j.sjbs.2021.01.052, PMID: 33911962 PMC8071897

[B107] Sun J. Li H. Wang Y. Du Z. Rengel Z. Zhang A. (2022). Biochar and nitrogen fertilizer promote rice yield by altering soil enzyme activity and microbial community structure. GCB. Bioenergy 14, 1266–1280. doi: 10.1111/gcbb.12995, PMID: 41834780

[B108] Sun H. Lu H. Chu L. Shao H. Shi W. (2017). Biochar applied with appropriate rates can reduce N leaching, keep N retention and not increase NH3 volatilization in a coastal saline soil. Sci. Tot. Environ. 575, 820–825. doi: 10.1016/j.scitotenv.2016.09.137, PMID: 27693153

[B109] Sun T. Yang W. Xu Y. Wang L. Liang X. Huang Q. . (2023). Effect of Ca–modified biochar coupling with low–Cd accumulation maize cultivars on remediation of Cd contaminated soils and microbial community composition. Soil Till. Res. 232, 105765. doi: 10.1016/j.still.2023.105765, PMID: 41853590

[B110] Suprasanna P. Nikalje G. C. Rai A. N. (2016). “ Osmolyte accumulation and implications in plant abiotic stress tolerance,” in Osmolytes and plants acclimation to changing environment: Emerging omics technologies ( Springer), 1–12. doi: 10.1007/978-81-322-2616-1_1, PMID:

[B111] Tian T. Wang J. Wang H. Cui J. Shi X. Song J. . (2022). Nitrogen application alleviates salt stress by enhancing osmotic balance, ROS scavenging, and photosynthesis of rapeseed seedlings (Brassica napus). Plant Signal. Behav. 17, 2081419. doi: 10.1080/15592324.2022.2081419, PMID: 35621189 PMC9154800

[B112] Tu C. Wei J. Guan F. Liu Y. Sun Y. Luo Y. (2020). Biochar and bacteria inoculated biochar enhanced Cd and Cu immobilization and enzymatic activity in a polluted soil. Environ. Int. 137, 105576. doi: 10.1016/j.envint.2020.105576, PMID: 32070805

[B113] Ullah S. Ali I. Liang H. Zhao Q. Wei S. Muhammad I. . (2021). An approach to sustainable agriculture by untangling the fate of contrasting nitrogen sources in double-season rice grown with and without biochar. Gcb. Bioenergy 13, 382–392. doi: 10.1111/gcbb.12789, PMID: 41834780

[B114] Van Tan L. Thanh T. (2021). The effects of salinity on changes in characteristics of soils collected in a saline region of the Mekong Delta, Vietnam. Open Chem. 19, 471–480. doi: 10.1515/chem-2021-0037, PMID: 41717541

[B115] Wang J. Liu H. Gong P. Li P. Yang C. Yang C. . (2025). Assessing the impact of residual film on agriculture: A meta-analysis of soil moisture, salinity, and crop yield. Ecotoxicol. Environ. Saf. 289, 117665. doi: 10.1016/j.ecoenv.2025.117665, PMID: 39793285

[B116] Wang J. Xiong Z. Kuzyakov Y. (2016). Biochar stability in soil: meta-analysis of decomposition and priming effects. Gcb. Bioenergy 8, 512–523. doi: 10.1111/gcbb.12266, PMID: 41834780

[B117] Wang R. Kang Y. Wan S. Hu W. Liu S. Liu S. (2011). Salt distribution and the growth of cotton under different drip irrigation regimes in a saline area. Agric. Water Manage. 100, 58–69. doi: 10.1016/j.agwat.2011.08.005, PMID: 41853590

[B118] Wang W. Zhang D. Kong H. Zhang G. Shen F. Huang Z. (2024). Effects of salinity accumulation on physical, chemical, and microbial properties of soil under rural domestic sewage irrigation. Agronomy 14, 514. doi: 10.3390/agronomy14030514, PMID: 41725453

[B119] Wang X. Li Y. Wang H. Wang Y. Biswas A. Chau H. W. . (2022). Targeted biochar application alters physical, chemical, hydrological and thermal properties of salt-affected soils under cotton-sugarbeet intercropping. Catena 216, 106414. doi: 10.1016/j.catena.2022.106414, PMID: 41853590

[B120] Wang Y. Wang H.-S. Tang C.-S. Gu K. Shi B. (2019). Remediation of heavy-metal-contaminated soils by biochar: a review. Environ. Geotech. 9, 135–148. doi: 10.1680/jenge.18.00091, PMID: 26962031

[B121] Waqas M. A. Kaya C. Riaz A. Farooq M. Nawaz I. Wilkes A. . (2019). Potential mechanisms of abiotic stress tolerance in crop plants induced by thiourea. Front. Plant Sci. 10, 1336. doi: 10.3389/fpls.2019.01336, PMID: 31736993 PMC6828995

[B122] Wei T. Li X. Li H. Gao H. Guo J. Li Y. . (2022). The potential effectiveness of mixed bacteria-loaded biochar/activated carbon to remediate Cd, Pb co-contaminated soil and improve the performance of pakchoi plants. J. Hazard. Mater. 435, 129006. doi: 10.1016/j.jhazmat.2022.129006, PMID: 35489314

[B123] Wen Y. Wu R. Qi D. Xu T. Chang W. Li K. . (2024). The effect of AMF combined with biochar on plant growth and soil quality under saline-alkali stress: Insights from microbial community analysis. Ecotoxicol. Environ. Saf. 281, 116592. doi: 10.1016/j.ecoenv.2024.116592, PMID: 38901167

[B124] Wu M. Li Y. Li J. Wang Y. Xu H. Zhao Y. (2019). Bioreduction of hexavalent chromium using a novel strain CRB-7 immobilized on multiple materials. J. Hazard. Mater. 368, 412–420. doi: 10.1016/j.jhazmat.2019.01.059, PMID: 30703702

[B125] Wu P. Wang Z. Bhatnagar A. Jeyakumar P. Wang H. Wang Y. . (2021). Microorganisms-carbonaceous materials immobilized complexes: Synthesis, adaptability and environmental applications. J. Hazard. Mater. 416, 125915. doi: 10.1016/j.jhazmat.2021.125915, PMID: 41853590

[B126] Wu P. Wang Z. Wang H. Bolan N. S. Wang Y. Chen W. (2020). Visualizing the emerging trends of biochar research and applications in 2019: a scientometric analysis and review. Biochar 2, 135–150. doi: 10.1007/s42773-020-00055-1, PMID: 41853694

[B127] Xing J. Li X. Li Z. Wang X. Hou N. Li D. (2024). Remediation of soda-saline-alkali soil through soil amendments: Microbially mediated carbon and nitrogen cycles and remediation mechanisms. Sci. Tot. Environ. 924, 171641. doi: 10.1016/j.scitotenv.2024.171641, PMID: 38471593

[B128] Xu Z. Shao T. Lv Z. Yue Y. Liu A. Long X. . (2020). The mechanisms of improving coastal saline soils by planting rice. Sci. Tot. Environ. 703, 135529. doi: 10.1016/j.scitotenv.2019.135529, PMID: 31759722

[B129] Xue Y. Su S. Wang X. Lin Z. (2017). Soil physical and chemical properties of five subtropical forests in lingao of hainan. Agric. Sci. Technol. 18, 1459–1464.

[B130] Yan G. Fan X. Peng M. Yin C. Xiao Z. Liang Y. (2020). Silicon improves rice salinity resistance by alleviating ionic toxicity and osmotic constraint in an organ-specific pattern. Front. Plant Sci. 11, 260. doi: 10.3389/fpls.2020.00260, PMID: 32226436 PMC7081754

[B131] Yao R. Gao Q. Liu Y. Li H. Yang J. Bai Y. . (2023). Deep vertical rotary tillage mitigates salinization hazards and shifts microbial community structure in salt-affected anthropogenic-alluvial soil. Soil Till. Res. 227, 105627. doi: 10.1016/j.still.2022.105627, PMID: 41853590

[B132] Yao Y. Sun Y. Feng Q. Zhang X. Gao Y. Ou Y. . (2021). Acclimation to nitrogen× salt stress in Populus bolleana mediated by potassium/sodium balance. Ind. Crops Prod. 170, 113789. doi: 10.1016/j.indcrop.2021.113789, PMID: 41853590

[B133] You J. Chan Z. (2015). ROS regulation during abiotic stress responses in crop plants. Front. Plant Sci. 6, 165102. doi: 10.3389/fpls.2015.01092, PMID: 26697045 PMC4672674

[B134] Yuan J.-H. Xu R.-K. Zhang H. (2011). The forms of alkalis in the biochar produced from crop residues at different temperatures. Bioresour. Technol. 102, 3488–3497. doi: 10.1016/j.biortech.2010.11.018, PMID: 21112777

[B135] Zhang B. Zhang J. Liu Y. Shi P. Wei G. (2018). Co-occurrence patterns of soybean rhizosphere microbiome at a continental scale. Soil Biol. Biochem. 118, 178–186. doi: 10.1016/j.soilbio.2017.12.011, PMID: 41853590

[B136] Zhang G. Bai J. Jia J. Wang W. Wang X. Zhao Q. . (2019a). Shifts of soil microbial community composition along a short-term invasion chronosequence of Spartina alterniflora in a Chinese estuary. Sci. Tot. Environ. 657, 222–233. doi: 10.1016/j.scitotenv.2018.12.061, PMID: 30543970

[B137] Zhang G. Bai J. Zhai Y. Jia J. Zhao Q. Wang W. . (2023). Microbial diversity and functions in saline soils: A review from a biogeochemical perspective. J. Adv. Res. 59, 129–140. doi: 10.1016/j.jare.2023.06.015, PMID: 37392974 PMC11081963

[B138] Zhang J. Bai Z. Huang J. Hussain S. Zhao F. Zhu C. . (2019b). Biochar alleviated the salt stress of induced saline paddy soil and improved the biochemical characteristics of rice seedlings differing in salt tolerance. Soil Till. Res. 195, 104372. doi: 10.1016/j.still.2019.104372, PMID: 41853590

[B139] Zhang J. Mason A. S. Wu J. Liu S. Zhang X. Luo T. . (2015a). Identification of putative candidate genes for water stress tolerance in canola (Brassica napus). Front. Plant Sci. 6, 1058. doi: 10.3389/fpls.2015.01058, PMID: 26640475 PMC4661274

[B140] Zhang M. Jin Z.-Q. Zhao J. Zhang G. Wu F. (2015b). Physiological and biochemical responses to drought stress in cultivated and Tibetan wild barley. Plant Growth Regul. 75, 567–574. doi: 10.1007/s10725-014-0022-x, PMID: 41853694

[B141] Zhang Y. Miao S. Song Y. Wang X. Jin F. (2024). Biochar application reduces saline–alkali stress by improving soil functions and regulating the diversity and abundance of soil bacterial community in highly saline–alkali paddy field. Sustainability 16, 1001. doi: 10.3390/su16031001, PMID: 41725453

[B142] Zhao Q. Bai J. Gao Y. Zhao H. Zhang G. Cui B. (2020). Shifts in the soil bacterial community along a salinity gradient in the Yellow River Delta. L. Degrad. Dev. 31, 2255–2267. doi: 10.1002/ldr.3594, PMID: 41848424

[B143] Zhao X. Bi G. Harkess R. L. Blythe E. K. (2016). Effects of different NH4: NO3 ratios on growth and nutrition uptake in Iris germanica ‘Immortality.’. HortScience 51, 1045–1049. doi: 10.21273/HORTSCI.51.8.1045

[B144] Zheng H. Wang Z. Deng X. Herbert S. Xing B. (2013). Impacts of adding biochar on nitrogen retention and bioavailability in agricultural soil. Geoderma 206, 32–39. doi: 10.1016/j.geoderma.2013.04.018, PMID: 41853590

