# Supplementary material for: Microbial-inoculated biochar combined with nitrogen mitigates salinity stress in rice by reducing salt accumulation and enhancing soil–plant interactions
Source: Front Plant Sci. 2026 Mar 24;17:1751156. doi: 10.3389/fpls.2026.1751156 (PMC13060037; doi:10.3389/fpls.2026.1751156)
Supplement: Supplementary file 1 [file DataSheet1.docx]

**Microbial inoculated biochar with nitrogen reduces salt accumulation in rice plants under salt-affected soils**

Hafiz Muhammad Mazhar Abbas^1^, Mohammad Nauman Khan^1^, Haider Sultan^1^, Asad Shah^1^, Fahd Rasul^2^, Ashar Tahir^3^, Muhammad Nafees-Ur-Rehman^4^, Lixiao Nie^1*^

^1^School of Breeding and Multiplication (Sanya Institute of Breeding and Multiplication), Hainan University, Sanya, 572000, China.

^2^Department of Agronomy, University of Agriculture Faisalabad, 38040, Faisalabad, Punjab, Pakistan

^3^School of Ecology and Environment, Hainan University, Haikou 570228, China.

^4^School of Life Sciences, Hainan University, Haikou 570228, China

^5^College of Forestry, Hainan University, Haikou 570228, China

Corresponding author: [lxnie@hainanu.edu.cn](mailto:lxnie@hainanu.edu.cn),

**This file include 4 Sections, 3 figures and 4 tables**

*Section 1. Microbial biochar preparation and characterization*

In this method, 10 grams of biochar were mixed in 100 ml microbial culture and placed in a shaking incubator (120 rpm) at 30 ℃ for one day. Once the bacteria and fungi were immobilized onto the biochar (BC), the composite materials were collected and freeze-dried. To distinguish between the two types, the resulting materials were named BB (for bacterial inoculated biochar) and BF for fungal inoculated biochar, respectively. The surface morphology of biochar (BC), bacteria-loaded biochar (BB) and fungal inoculated biochar (BF) were analyzed using a Scanning electron microscope (German, Zeiss Sigma 300), XRD (JP Rigaku Smart Lab SE), EDS (OxfordX-MAX) and FTIR (USA, Thermo Scientific iN 10).

*Section 2. Survival of free and biochar (BC) immobilized bacteria in salt-contaminated soil*

One milliliter each of bacterial and fungal cultures, or one gram of microbial-loaded material, were thoroughly mixed with 50 mL of sterile water using a vortex mixer. Following serial dilution of the suspension, 100 µL aliquots were spread onto LB agar plates. The Petri dishes containing LB agar (LB plates) were incubated for 24 hours at 30 ℃. Afterward, we counted the number of colonies that grew on each plate. This allowed us to calculate the number of viable (living) bacteria in 1 milliliter (mL) of *Mycobacterium sp*. and *Penicillium sp*. in each solution and 1 gram of each composite material. To ensure equivalent viable microbes across treatments, 1 mL each of *Mycobacterium sp*. and *Penicillium sp*. (2 × 10¹⁰ CFU mL⁻¹), or 0.48 g of biochar loaded with fungi (BF, 3.9 × 10¹⁰ CFU g⁻¹) or 0.3 g of biochar loaded with bacteria (BB, 4.5 × 10¹⁰ CFU g⁻¹), were added to 10 g of sterilized soil and incubated in the dark at 30 °C. The soil was treated with saline water with 0.4% NaCl solution and was moistened to 60% of its capacity to hold water and this moisture level was maintained by adding small amounts of distilled water regularly. To track changes over time, we took equal amounts of the soil sample at 0 days, 10 days, 20 days, 30 days, and 40 days. We used the same method mentioned earlier to count the number of living microbes (CFU g^-1^) in the soil samples.

## *Section 3. Chemical properties of post-harvest soil*

Samples were collected manually from the rhizosphere from selected treatments. The soil samples were first air-dried and then cleaned by removing any stones or leftover plant debris (residual litter). Once dry and cleaned, the soil was ground up to a fine consistency, able to pass through a sieve with holes 2 millimeters wide. Finally, this prepared soil was stored for further analysis. A smaller portion of the air-dried soil that already passed through a 2-millimeter sieve was further ground even finer. This finer fraction, able to pass through a sieve with holes only 0.15 millimeters wide, was then used for the following soil analyses. Walkley-Black method was used to measure organic matter (FAO 1974; Walkley 1947). The pH of soil was measured through a mixture of (1:2) soil-water by using a pH meter. Soluble Na^+^ and K^+^ contents were measured in accordance with the method by a flame photometer method. Ammonium nitrogen (NH_4_^+^-N) and nitrate nitrogen (NO_3_^-^-N) were quantified using the indophenol blue method (Maynard, Kalra, and Crumbaugh 1993).

*Section 4. Relative water content and membrane stability index*

Fresh leaf samples (0.5 g) were weighed using an HR-60 precision balance and then submerged in water for 4 hours in day time to reach turgor. The leaves were then oven-dried at high temperature until a constant weight was achieved. The RWC was calculated using the formula provided below.

RWC (%) = [((FW-DW)) ⁄ ((TW-DW)] ×100)

To measure MSI, 50 mg of leaf material was weighed out in duplicate and placed in test tubes containing 10 mL of double-distilled water. Further, one set of samples was incubated in a water bath at 40°C for 30 minutes (C1). The electrical conductivity of the solution was measured using a CL-250 conductivity meter on a conductivity bridge. Similarly, the second set of samples was boiled in a water bath for 10 minutes (C2), and their conductivity was measured using the conductivity bridge. The MSI was calculated using the formula provided below,

MSI (%) = [1- (C1/C2)] ×100


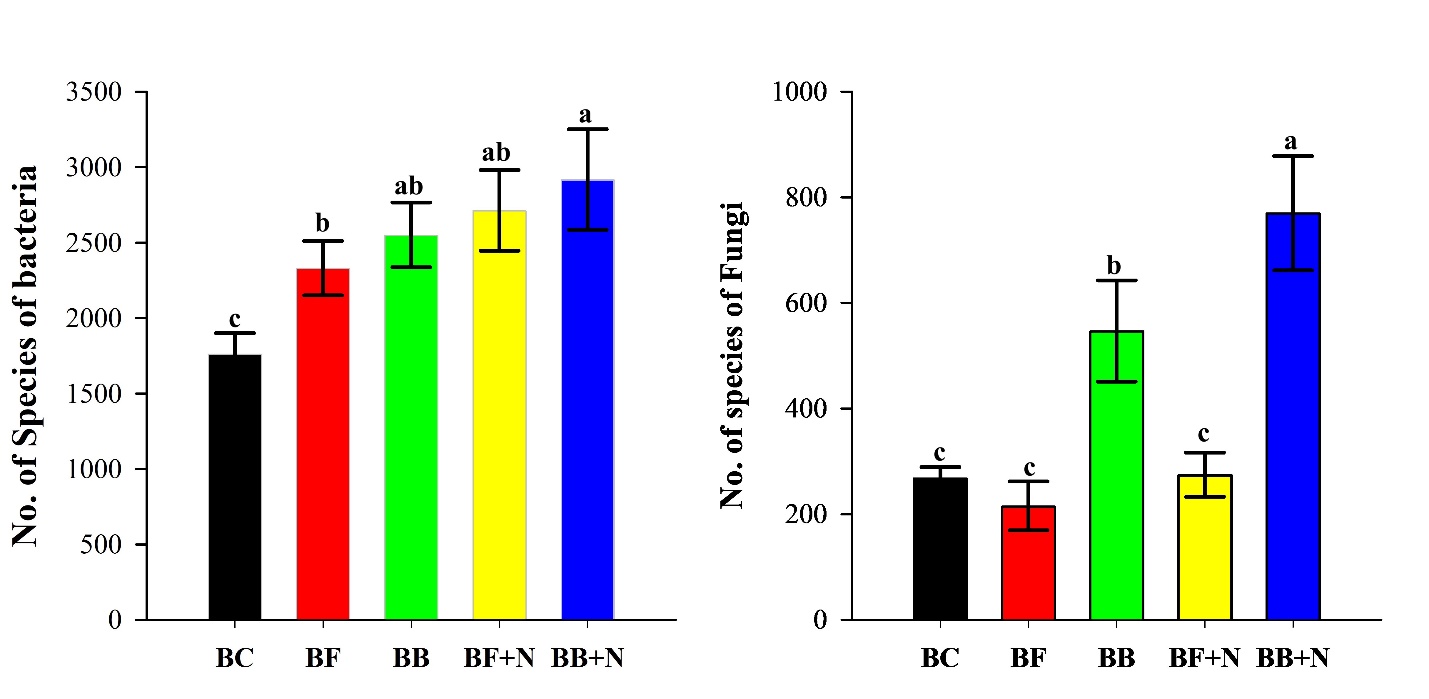


**Figure S1.** No. of species of bacteria (A) and Fungi (B) in different treatments


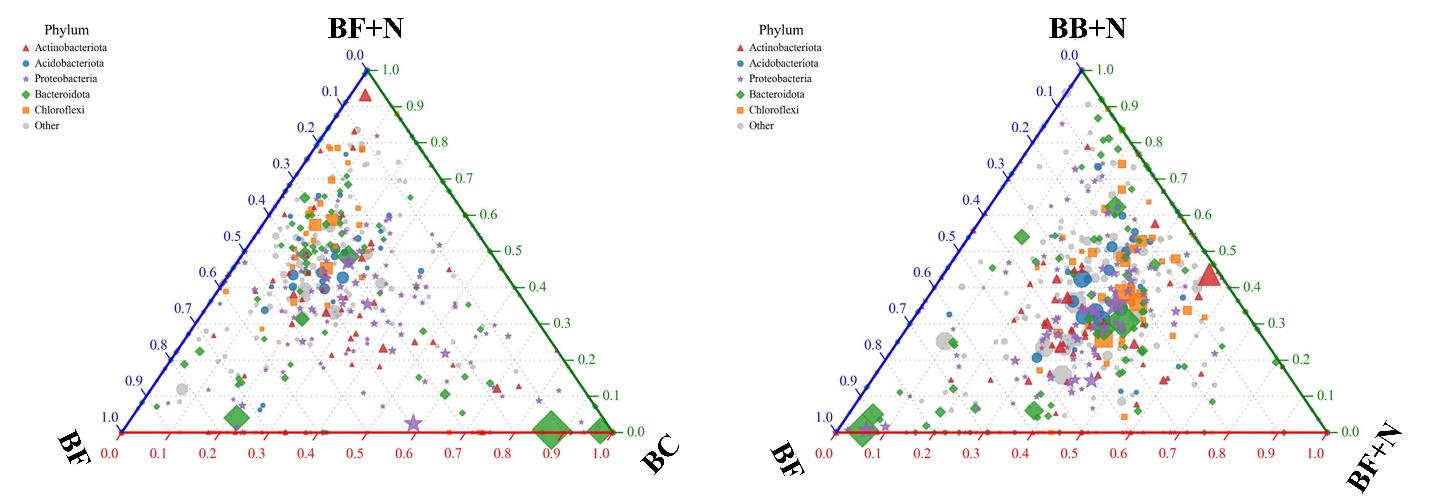


**Figure S2.** Relative abundance of actinobacteria. Proteobacteria and chlorofexi in saline conditions.


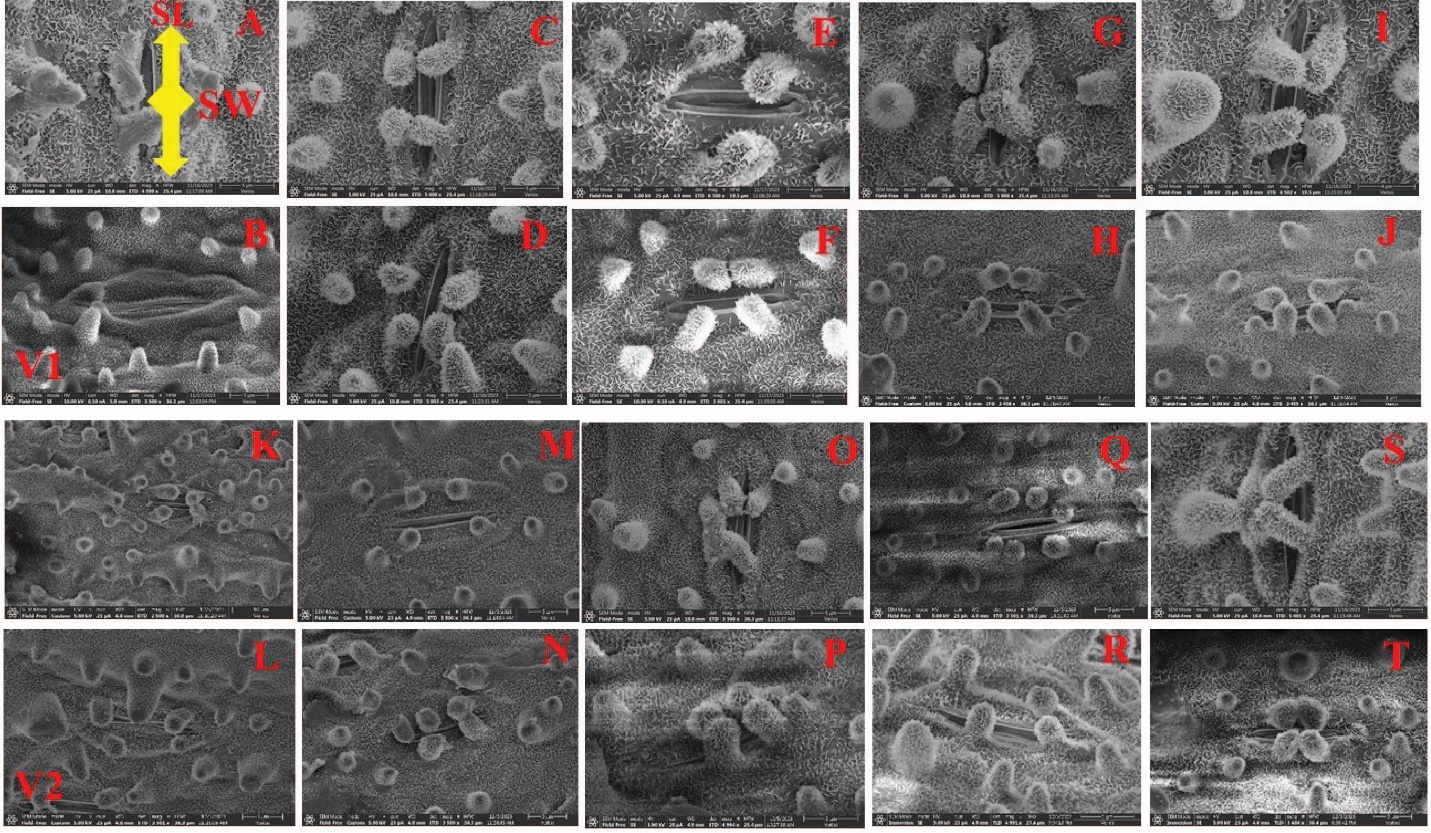


**Figure S3**. SEM images of stomata of two rice varieties (V1= SLY138, V2= JLY534) grown under synergistic effect of microbial inoculated biochar and N fertilizer. For SLY138 (A) BC non-saline conditions (B) BC saline conditions (C) BF for non-saline conditions (D)BF saline conditions (E) BB for non-saline conditions (F) BB saline conditions (G) BF + N120 for non-saline conditions (H) BF + N120 saline conditions (I) BB + N120 for non-saline conditions (J) BB + N120 saline conditions. For JLY534 (K) BC non-saline conditions (L) BC saline conditions (M) BF for non-saline conditions (N)BF saline conditions (O) BB for non-saline conditions (P) BB saline conditions (Q) BF + N120 for non-saline conditions (R) BF + N120 saline conditions (S) BB + N120 for non-saline conditions (T) BB + N120 saline conditions.

**Table S1:** Pre-experimental properties of soil

|  | Total Nutrient Content (g/kg) | | | |  | | | Available Nutrient Content (mg/kg) | | | | |
| --- | --- | --- | --- | --- | --- | --- | --- | --- | --- | --- | --- | --- |
| pH | OM | TN | TP | TK | Moisture content % | CEC cmol (+)/kg | EC (µs/cm) | AP | AK | Nitrate N | Ammonium N | |
| 6.51 | 1.05 | 0.13 | 0.21 | 0.65 | 2.57 | 2.14 | 267 | 4.49 | 27.35 | 0.95 | 1.87 |  |

**Table S2:** pH and nutrient status of biochar

| pH | 9 |
| --- | --- |
| TC (At%) | 80 |
| N (At%) | 2.45 |
| O (At%) | 1 |
| P (At%) | 0.09 |
| K (At%) | 0.14 |

**Table S3.** Treatment Plan for experiment

| **Treatments** | **salt levels** | **Treatment Name** | **Application Rates** |
| --- | --- | --- | --- |
| T1 | 0% | N60 | Nitrogen @ 60 kg ha^-1^ |
|  | 0.40% | N60 | Nitrogen @ 60 kg ha^-1^ |
| T2 | 0% | N120 | Nitrogen @ 120 kg ha^-1^ |
|  | 0.40% | N120 | Nitrogen @ 120 kg ha^-1^ |
| T3 | 0% | BC | Simple Rice Straw biochar (1%) |
|  | 0.40% | BC | Simple Rice Straw biochar (1%) |
| T4 | 0% | BF | Fungal Inoculated biochar (1%) |
|  | 0.40% | BF | Fungal Inoculated biochar (1%) |
| T5 | 0% | BB | Bacterial Inoculated biochar (1%) |
|  | 0.40% | BB | Bacterial Inoculated biochar (1%) |
| T6 | 0% | BC+N60 | Simple Rice Straw biochar (1%)+Nitrogen @ 60 kg ha-^1^ |
|  | 0.40% | BC+N60 | Simple Rice Straw biochar (1%)+Nitrogen @ 60 kg ha^-1^ |
| T7 | 0% | BC+N120 | Simple Rice Straw biochar (1%)+Nitrogen @ 120 kg ha^-1^ |
|  | 0.40% | BC+N120 | Simple Rice Straw biochar (1%)+Nitrogen @ 120 kg ha^-1^ |
| T8 | 0% | BF+N60 | Fungal Inoculated biochar (1%)+Nitrogen @ 60 kg ha^-1^ |
|  | 0.40% | BF+N60 | Fungal Inoculated biochar (1%)+Nitrogen @ 60 kg ha^-1^ |
| T9 | 0% | BF+N120 | Fungal Inoculated biochar (1%)+Nitrogen @ 120 kg ha^-1^ |
|  | 0.40% | BF+N120 | Fungal Inoculated biochar (1%)+Nitrogen @ 120 kg ha^-1^ |
| T10 | 0% | BB+N60 | Bacterial Inoculated biochar (1%)+Nitrogen @ 60 kg ha^-1^ |
|  | 0.40% | BB+N60 | Bacterial Inoculated biochar (1%)+Nitrogen @ 60 kg ha^-1^ |
| T11 | 0% | BB+N120 | Bacterial Inoculated biochar (1%)+Nitrogen @ 120 kg ha^-1^ |
|  | 0.40% | BB+N120 | Bacterial Inoculated biochar (1%)+Nitrogen @ 120 kg ha^-1^ |

**Table S4.** Selected treatments on the base of best performance in morphology of rice plants

| Treatments | salt levels | Treatment Name | Application Rates |
| --- | --- | --- | --- |
| T3 | 0% | BC | Simple Rice Straw biochar (1%) |
|  | 0.40% | BC | Simple Rice Straw biochar (1%) |
| T4 | 0% | BF | Fungal Inoculated biochar (1%) |
|  | 0.40% | BF | Fungal Inoculated biochar (1%) |
| T5 | 0% | BB | Bacterial Inoculated biochar (1%) |
|  | 0.40% | BB | Bacterial Inoculated biochar (1%) |
| T9 | 0% | BF+N120 | Fungal Inoculated biochar (1%)+Nitrogen @ 120 kg ha^-1^ |
|  | 0.40% | BF+N120 | Fungal Inoculated biochar (1%)+Nitrogen @ 120 kg ha^-1^ |
| T11 | 0% | BB+N120 | Bacterial Inoculated biochar (1%)+Nitrogen @ 120 kg ha^-1^ |
|  | 0.40% | BB+N120 | Bacterial Inoculated biochar (1%)+Nitrogen @ 120 kg ha^-1^ |

**References**

FAO. 1974. “The Euphrates Pilot Irrigation Project. Methods of Soil Analysis. Gadeb Soil Laboratory (a Laboratory Manual).”

Maynard, D. G., Y. P. Kalra, and J. A. Crumbaugh. 1993. “Nitrate and Exchangeable Ammonium Nitrogen.” *Soil Sampling and Methods of Analysis* 1:25–38.

Walkley, Allan. 1947. “A Critical Examination of a Rapid Method for Determining Organic Carbon in Soils—Effect of Variations in Digestion Conditions and of Inorganic Soil Constituents.” *Soil Science* 63(4):251–64.
